# How 5 *f* Electron Polarisability Drives Covalency and Selectivity in Actinide *N*‐Donor Complexes

**DOI:** 10.1002/chem.202102849

**Published:** 2021-12-02

**Authors:** Luisa Köhler, Michael Patzschke, Moritz Schmidt, Thorsten Stumpf, Juliane März

**Affiliations:** ^1^ Helmholtz-Zentrum Dresden–Rossendorf (HZDR) Institute of Resource Ecology Bautzner Landstraße 400 01328 Dresden Germany

**Keywords:** actinides, bonding analysis, *f*-electrons, *N*-donor ligand, pyrrole

## Abstract

We report a series of isostructural tetravalent actinide (Th, U−Pu) complexes with the *N*‐donor ligand *N*,*N’*‐ethylene‐bis((pyrrole‐2‐yl)methanimine) (H_2_ **L**, H_2_pyren). Structural data from SC‐XRD analysis reveal [An(pyren)_2_] complexes with different An−N_imine_ versus An−N_pyrrolide_ bond lengths. Quantum chemical calculations elucidated the bonding situation, including differences in the covalent character of the coordinative bonds. A comparison to the intensely studied analogous *N,N′‐*ethylene‐bis(salicylideneimine) (H_2_salen)‐based complexes [An(salen)_2_] displays, on average, almost equal electron sharing of pyren or salen with the An^IV^, pointing to a potential ligand‐cage‐driven complex stabilisation. This is shown in the fixed ligand arrangement of pyren and salen in the respective An^IV^ complexes. The overall bond strength of the pure *N*‐donor ligand pyren to An^IV^ (An=Th, U, Np, Pu) is slightly weaker than to salen, with the exception of the Pa^IV^ complex, which exhibits extraordinarily high electron sharing of pyren with Pa^IV^. Such an altered ligand preference within the early An^IV^ series points to a specificity of the 5*f* ^1^ configuration, which can be explained by polarisation effects of the 5 *f* electrons, allowing the strongest *f* electron backbonding from Pa^IV^ (5*f* ^1^) to the *N* donors of pyren.

## Graphical abstract

**A series of isostructural actinide (Th, U−Pu) complexes** with *N*,*N’*‐ethylene‐bis((pyrrole‐2‐yl)methanimine) (H_2_ **L**, H_2_pyren) reveal different An−N_imine_ vs. An−N_pyrrolide_ bond lengths and covalent character of the An−N bonds. Comparison to the [An(salen)_2_] complexes displays a slightly weaker overall bond strength of pyren to An^IV^ (An=Th, U, Np, Pu), except for Pa^IV^.

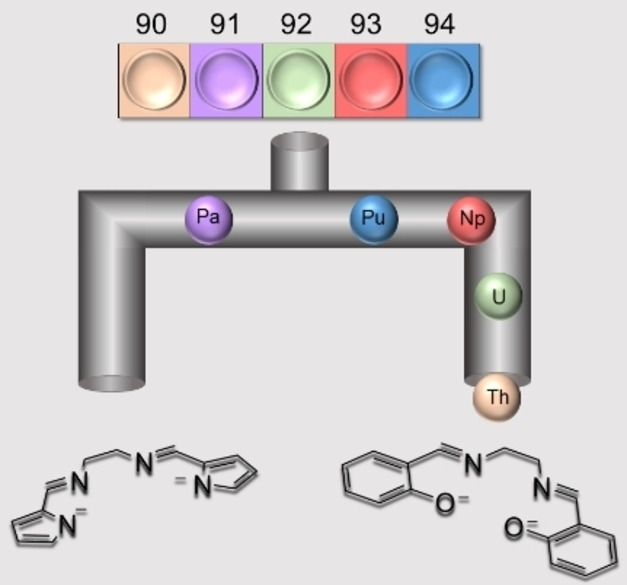

## Introduction

The characteristic 5 *f* shell of the actinides not only determines their magnetic and spectroscopic properties, but significantly participates in chemical bonding especially for early actinides (U−Pu).[^1^, ^2^] The stability of the resulting bond is then determined by the orbital overlap (in space and energy), which itself depends on the electronic situation at the actinide (i. e., element, oxidation state) and the ligand's donor atoms and their chemical environment. One intriguing example is the pure *N*‐donor ligands of BTP (bistriazinyl pyridine) or BTBP (bistriazinyl bipyridine) type, which were found to be selective for An^III^ over Ln^III^. Many experimental and theoretical studies to elucidate the origin of this selectivity have not come to a consensus so far.[^2^, ^3^, ^4^, ^5^] In part motivated by such selectivity, multiple studies have attempted to delineate trends in An−N bonding as a function of for example oxidation state and *N*‐donor functionality. For the tetravalent oxidation state, Huang et al. performed theoretical investigations on the [An(OPh)_3_]_2_(*μ*–*η* ^2^:*η* ^2^‐N_2_) (An=Th−Pu) system. Traversing the actinide series they found the Pa^IV^ complex to exhibit the greatest An→N_2_ backbonding effect, correlating with smallest, calculated N−N stretching wavenumber and An−N bond distance. Molecular orbital analysis shows the greatest nitrogen contribution for the Pa case, confirming backbonding as the explanation for the observed phenomenon. For the following actinides U−Pu the backbonding is less pronounced and changes very little between the elements.^[6]^ Most recently, a similar trend was also observed in complexes of the mixed *N*,*O*‐donor [M(salen)_2_] (salen=*N*,*N’*‐bis(salicylidene)‐ethylenediamine, M=Ce, Th−Pu). A prominent backbonding effect from Pa to the ligand‐based orbitals leads to enhanced bond strength and increased covalent character in [Pa(salen)_2_], whereas for the other metal centres similar bond characteristics could be observed.^[7]^

However, in the salen system An bonding was dominated by the strong, hard *O*‐donor, which ultimately limits the flexibility of the An−N bond to react to changes in electronic situation, for example, caused by backbonding. Studies of pure N donors remain scarce, especially for the transuranium elements.[^8^, ^9^, ^10^, ^11^] To the best of our knowledge, only two Pu^IV^ complexes exclusively stabilised by *N*‐donor ligands are known in the literature: One containing the inorganic NCS^−^ ligands^[12]^ and one with a TREN (tris(2‐aminoethyl)amine)‐type ligand.^[13]^

To address the question of An−N (back)bonding in metal–organic actinide compounds and expand the structural database of transuranium element complexes, we here study the actinide complex series of the pure *N*‐donor ligand *N*,*N’*‐ethylene‐bis((pyrrole‐2‐yl)methanimine (H_2_pyren, H_2_ **L**, which is structurally closely related to salen. However, it only contains two different nitrogen atoms as binding partners, one of them in a heterocyclic environment possibly relevant for minor actinide separations technologies. According to Pearson's concept of hard and soft acids and bases An^IV^ ions should interact strongly with hard bases, raising the question how a negatively charged nitrogen would behave as binding partner compared to the neutral imine *N*‐donor and how this changes with increasing hardness over a series of early actinides.^[14]^ Due to the elements’ decreasing ionic radii one would expect the hardness to increase from Th to Pu, on the other hand, multidentate ligands form a relatively rigid scaffolding which might be more difficult to fill for continuously smaller actinide ions. All compounds, including the transuranium complexes with Np and Pu, were thoroughly characterised in the solid state by single‐crystal X‐ray diffractometry (SC‐XRD) and IR spectroscopy. The experimental data were then completed by characterisation in solution (NMR and UV/Vis spectroscopy) and complemented by in‐depth quantum chemical investigations at the density functional theory (DFT) and multireference level to obtain the desired electronic structure information.

## Results and Discussion

**Scheme 1:**
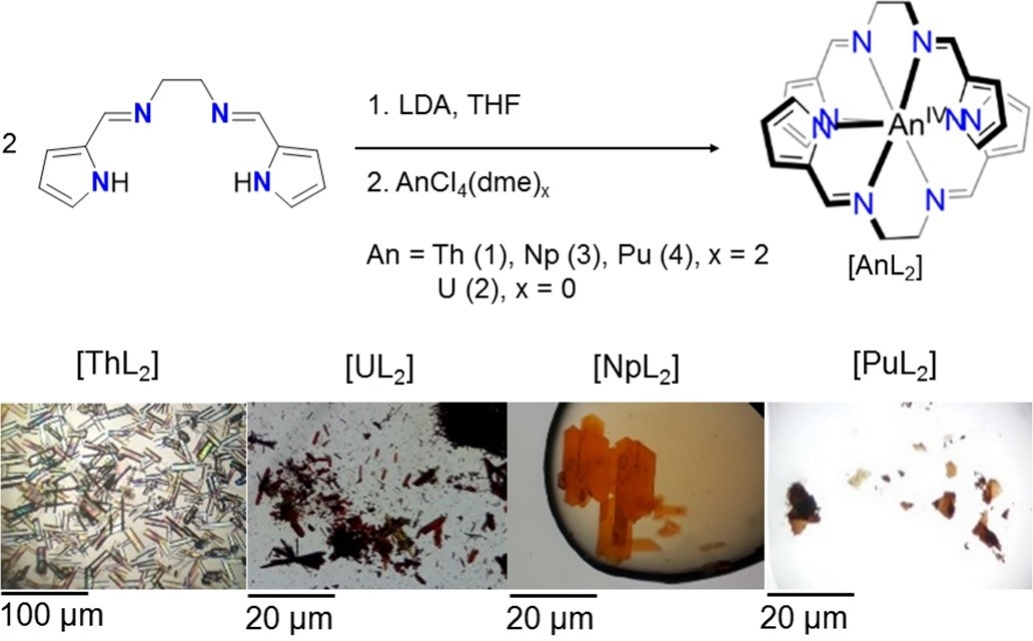
Top: two‐step synthesis of the complexes [An**L**_2_] (An=Th, U−Pu), and bottom: crystal pictures.

Ligands were synthesised according to a literature procedure in a condensation reaction.^[15]^ The complex was synthesised according to a two‐step route, in which the ligand deprotonation with the non‐coordinative base lithium diisopropylamide (LDA) in the first step is followed by a salt metathesis reaction with the actinide chloride in case of uranium and the actinide chloride dimethoxyethane (dme) adduct for Th, Np, Pu. Upon introduction of the tetravalent actinide source in the second step, the bis‐*N*,*N’*‐ethylene‐bis((pyrrole‐2‐yl)methanimine actinide ([An**L**_2_], An=U−Pu) complexes **2**–**4** precipitated as microcrystalline solids (Scheme 1). As complex **1** (bis‐*N*,*N’*‐ethylene‐bis((pyrrole‐2‐yl)methanimine thorium [Th**L**_2_]) did not precipitate, the volatile components were removed under vacuum to yield the solid product. Recrystallisation of complexes **1**–**4** from hot acetonitrile (MeCN) yielded crystals suitable for SC‐XRD. The analogous Pa complex could not be synthesised due to a lack of sufficient starting material.

**Figure 1:**
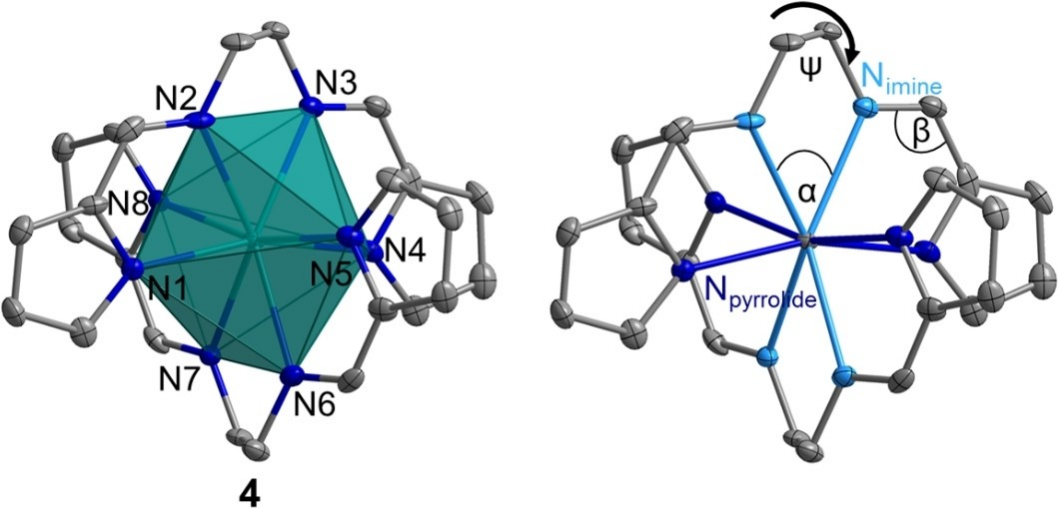
Molecular structure of [Pu(pyren)_2_] (**4**) with a coordination polyhedron, representing the isostructural complex series **1**–**4** (left) and different *N*‐donor entities visualised (right). Colour code (left): nitrogen: blue, carbon: grey, plutonium: petrol; right: imine N atoms: light blue, pyrrolide N atoms: dark blue. Co‐crystallised MeCN solvent molecules and hydrogen atoms are omitted for clarity. Ellipsoids are drawn at the 50 % probability level.

The actinide complexes crystallise isostructurally in the space group *Pca*2_1_ with similar cell parameters. Two doubly deprotonated ligand molecules coordinate the metal centre in a pincer‐type fashion, leading to an overall neutral complex. As expected, this is analogous to the already described bissalen complexes [An(salen)_2_] (An=Th, U−Pu).^[7]^ In **1**–**4**, one complex molecule and two additional acetonitrile solvent molecules form the asymmetric unit. All four available *N*‐donor atoms of each ligand bind to the An, which leads to an eightfold coordination, creating a polyhedron best described as a snub disphenoid (Johnson solid J_84_; Figure 1).

The isostructurality of the reported [An**L**_2_] (An=Th, U−Pu) complexes is further proven by their very similar IR spectra (see the Supporting Information). The prominent band at 1580 cm^−1^ for all complexes **1**–**4** can be assigned to the imine‐stretching mode. Finding the C=N stretching mode at the same wave number for all compounds could suggest that bonding to this moiety is identical for all actinides. A direct link between the vibrational frequency and bond strength for An−N bonds was however recently questioned in the context of tetravalent azido complexes.^[8]^ If the recorded spectra cannot reflect potential changes in ligand bond strength to varying actinide centres, they might rather be indicative of the formation of a fairly rigid ligand cage.

An analysis of the An−N bonds reveals a continuous decrease due to the decreasing ionic radii of the An^IV^. In all complexes **1**–**4** the averaged bond lengths An−N_pyrrolide_ are 0.06–0.08 Å shorter than the corresponding An−N_imine_ bond (Table 1). This cannot be exclusively explained by the charge differences of the negatively charged N_pyrrolide_ compared to the neutral N_imine_, because the conjugated system delocalises the electron density over both *N*‐donor moieties. This was confirmed by our DFT optimisations of the complexes, where an effective charge of approximately −0.5 can be found at each *N*‐donor entity (Table 2). However, the lone pair at N_pyrrolide_ enables a better overlap with the An's orbitals compared to N_imine_, leading to a shorter bond distance. Interestingly, the difference between the An−N_pyrrolide_ and An−N_imine_ bond lengths increases continuously with decreasing ionic radius from Th to Pu. This indicates a steric hindrance within the ligand, arising from its partial conjugation. The pyrrolide ring and the imine entity form a relatively rigid conjugated system, proved by the relevant angles *α* and *β* (Figure 1) of approximately 120° and 63°, respectively, for all compounds (see the Supporting Information). Adjustment to the actinide ion's size can then only occur by a relatively minor change in the torsion angle of the ethylene bridge *ψ* from 43° in **1** to 40° in **4**. Eventually, this leads the pyrrolide moieties to arrange closer to the An with decreasing ionic radius, while the imines in the bridge are relatively fixed in place. This is confirmed by a QTAIM analysis, where an increasing deviation of the An‐N_imine_ bond path from linearity is observed.

**Table 1:** Averaged bond lengths of complexes **1**–**4**.

Complex	Bond length [Å]			
	i.r. (CN=8)^[16]^	An−N_pyrrolide_	An−N_imine_	Δ^[a]^
1, [ThL_2_]	1.05	2.503 (20)	2.560 (28)	0.057
2, [UL_2_]	1.00	2.438 (3)	2.517 (3)	0.079
3, [NpL_2_]	0.98	2.423 (6)	2.503 (6)	0.08
4, [PuL_2_]	0.96	2.407 (6)	2.490 (8)	0.083

[a] Calculated difference between An−N_pyrrolide_ and An−N_imine_ bonds.

**Table 2:** NBO‐based natural population analysis for the free ligand **L** and the [An(pyren)_2_] (An=Th−Pu) complexes.

	NPA				
	*d* population	*f* excess	*q*(An)	*q*(N_pyrrolide_)	*q*(N_imine_)
**L**	–	–	–	−0.56	−0.50
Th	1.03	0.71	2.04	−0.58	−0.54
Pa	1.08	0.77	1.93	−0.56	−0.52
U	1.12	0.85	1.81	−0.54	−0.50
Np	1.12	0.86	1.79	−0.54	−0.51
Pu	1.09	0.85	1.81	−0.54	−0.50

Comparison of the experimentally determined An−N bond distances with literature shows that the An−N_pyrrolide_ and An−N_imine_ bond lengths in **1**–**4** are at the very bottom or even below the reported range in similar coordination environments. This is more pronounced for the An−N_pyrrolide_ bond than for the An−N_imine_ bonds. The greatest deviation between the shortest hitherto reported bond and the bond length determined for [An**L**_2_] is found in the Th complex **1**. Remarkably, the Th−N_pyrrolide_ bond in [Th**L**_2_] is more than 0.20 Å shorter than in a diphosphazide complex published by Dickie et al. (2.503 vs. 2.709 Å).^[17]^ The An−N_pyrrolide/imine_ bond lengths for the U, Np, and Pu complexes are still 0.06–0.07 Å shorter than the shortest literature values.[^18^, ^19^, ^20^, ^21^, ^22^, ^23^] A Pu bond to a pyrrolide nitrogen has never been reported, which makes the Pu−N_pyrrolide_ bond length with 2.407 Å a new addition to the Pu−N bond lengths data set. Based on the observations made for all other An−N bonds in this pyren system, we can nonetheless assume that this bond is on the short end of Pu−N_pyrrolide_ bonds once other systems will be characterised.

**Figure 2:**
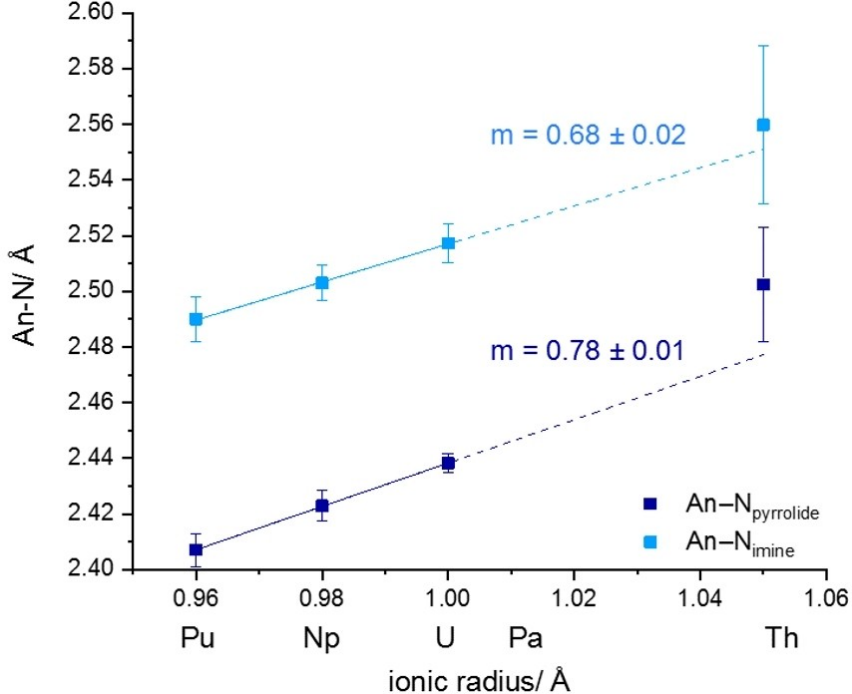
Linear regression (—) and extrapolation (‐ ‐ ‐ ‐) of An−N_pyrrolide_ (dark blue) and An−N_imine_ (light blue) bond lengths plotted against the tetravalent metal ionic radii. *R* ^2^=0.99 for both linear regressions. Error bars correspond to the simple standard deviation.

Plotting the An−N_pyrrolide_ and An−N_imine_ bond lengths against the ionic radii of Th−Pu, linear trends for both An−N bonds (*R* ^2^=0.99) can be observed from U−Pu (Figure 2).^[16]^ The slope of the linear regression for An−N_pyrrolide_ is with 0.78 (**1**) significantly larger than for the An−N_imine_ regression 0.68 (**2**), underpinning a stronger adaptation to the changed ionic radius with the An−N_pyrrolide_ bond than An−N_imine_ bond.

For both Th−N bonds the determined bond distances are too long to fit into the extrapolated linear regression of the other An−N lengths (dashed line in Figure 2). This indicates a weaker bond for the Th complex compared to the other actinides, albeit the Th^IV^ cation's size matches the binding pocket of pyren very well. Moreover, the linear trends in Figure 2 indicate the expected predominantly ionic bonds An−N_pyrrolide_ and An−N_imine_.^[7]^ A more profound investigation of the An−N bond character can however only be based on additional quantum chemical data.

### Computational results

To assess the bonding situation in the [An**L**_2_] complexes and eventually link the findings from structural analysis to bond characteristics, quantum chemical calculations on a DFT level were performed now also including [Pa**L**_2_].

In an initial step the structures of all complexes as well as the free, deprotonated ligand were optimised. The free ligand shows the expected geometry, with a small charge difference between the two *N*‐donor moieties of 0.06 (i. e., −0.56 and −0.50 for the pyrrolidine and the imine N, respectively). In the geometry‐optimised complexes, the ligand molecules occupy a highly symmetrical pincer‐type coordination environment and display similar bond lengths compared to the experimentally determined structures with a maximum deviation of only 0.012 Å for the Th−N_pyrrolide_ bond in **1** (see the Supporting Information). An NBO analysis of the optimised structures shows an even greater equalisation of the nitrogen charges in the complex than in the free ligand, of only 0.04 or even 0.03 for Np. As expected the pyrrolidine nitrogen is the slightly more negatively charged site in all cases. In the free salen ligand, the difference in charges is much larger (−0.44 for N and −0.78 for O) which is then reduced to −0.54 and −0.70 in the uranium complex. This larger difference is mainly due to differences in the electronegativity of N and O and partly due to the placement of the phenyl oxygen outside of the aromatic ring. In contrast to the salen system, charge differences can thus be expected to have negligible impact on An bonding in the pyren system.

We can evaluate electron density differences between free and bound components to obtain a first qualitative picture of the bonding situation (see the Supporting Information). All plots display an increase in electron density between the An centre and the *N*‐donors. Its rotational symmetric shape indicates the formation of a σ bond, which is expected for N as σ‐donor atom. Interestingly, for the Th complex the increase is much less pronounced compared to complexes with the other An Pa−Pu. This can be attributed to the presence of *f*‐electrons in the complexes with Pa−Pu and their involvement in bond formation, suggesting *f*‐electron backbonding.

From the NBO analysis of the complexes, it becomes apparent that Th shows the most ionic bond with the highest metal charge and lowest *f* excess and *d* population. The *f* excess population is the number of *f*‐electrons in excess of the expected value for An^IV^.

The *f* excess and *d* population then increase to Pa^IV^, while the metal's charge decreases, clearly indicating an increase in bond strength and covalency when *f*‐electrons are available to participate in bonding. U^IV^ shows yet higher orbital populations and lower charge than Pa^IV^, while the transuranium elements Np and Pu exhibit remarkably little change from U^IV^’s values.

**Figure 3:**
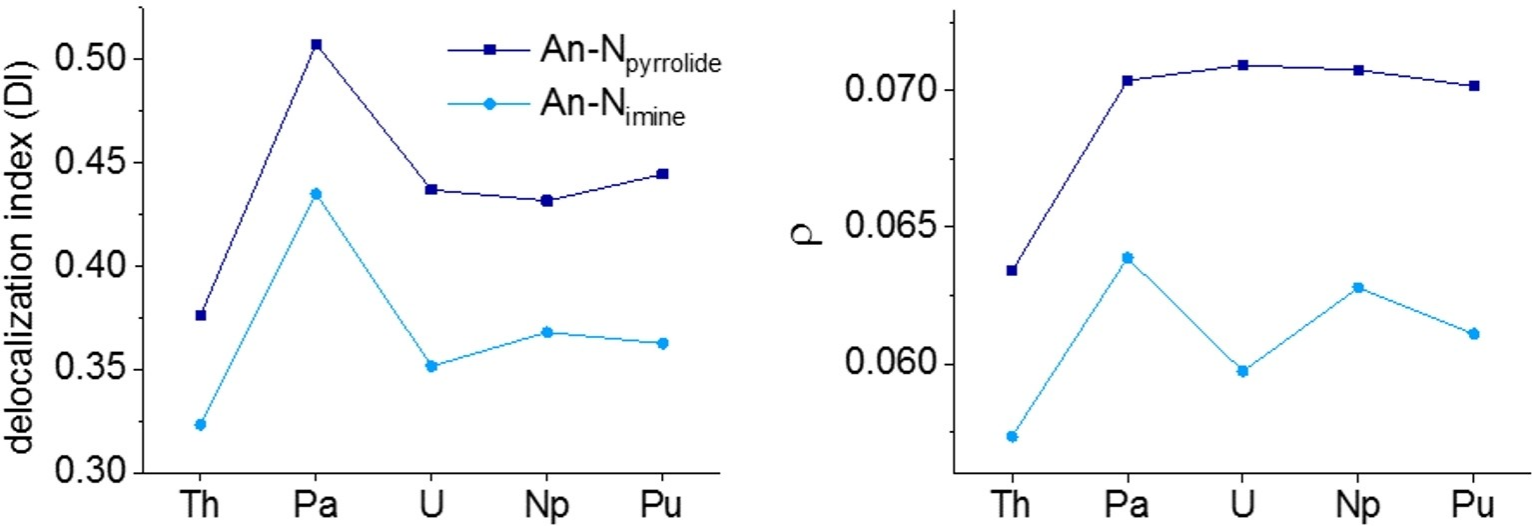
Values for the delocalisation index (left) and *ρ* (right) for the An−N_pyrrolide_ and An−N_imine_ bonds in [An(pyren)_2_] complexes (An=Th−Pu).

By making use of Bader's quantum theory of atoms in molecules (QTAIM) as well as natural population analysis (NPA) of the optimised structures, a detailed picture of the bonding situation can be drawn. The delocalisation index (DI) and the electron density at the bond critical point (*ρ*) are parameters to describe the bond characteristics. In both cases, a larger value corresponds to a greater strength and covalent character of the chemical bond. Comparing the two *N*‐donor moieties, the DI and *ρ* values for the An−N_pyrrolide_ bonds consistently exceed those of the An−N_imine_ bonds on average by 0.071 and 0.008, respectively (Figure 3). This clearly proves a more covalent character in the An−N_pyrrolide_ bonds than in the An−N_imine_ bonds as previously indicated by shorter experimental and calculated bond distances.

Overall, Th exhibits the smallest values for the An−N_imine_ bond at 0.32 and 0.057 when compared to the average values for the complexes containing U−Pu, which form a plateau at 0.36 (DI) and 0.061 (*ρ*), indicating a similar bonding situation. The same picture arises for the An−N_pyrrolide_ bond, where Th is at a minimum of 0.38 (DI) and 0.063 (*ρ*) compared to the plateau formed by U−Pu complexes at 0.44 (DI) and 0.071 (*ρ*). Therefore, Th exhibits the weakest An−N bonds, which can be explained by the lack of *f*‐electron backbonding. Remarkably, unexpectedly high DI values for the Pa complex of 0.51 (Pa−N_pyrrolide_) and 0.43 (Pa−N_imine_) break the observed trend.

The unexpected DI values for the Pa complex led us to perform a series of multireference calculations in order to ascertain the validity of our DFT‐based analysis. The details of these calculations can be found in the Supporting Information. The results show that our DFT‐based approach is valid and we will use these values throughout the manuscript.

The special bonding situation for the Pa−N bonds arises from the single *f*‐electron of Pa^IV^, leading to diffuse and highly polarisable *f‐*orbitals, which would be exceptionally well suited for backbonding. With decreasing ionic radius, the *f*‐orbitals then become more contracted and less polarisable, diminishing the orbital overlap and thus reducing the extent of *f*‐electron backbonding from U onwards.

### Comparison to [An(salen)_2_]

**Figure 4:**
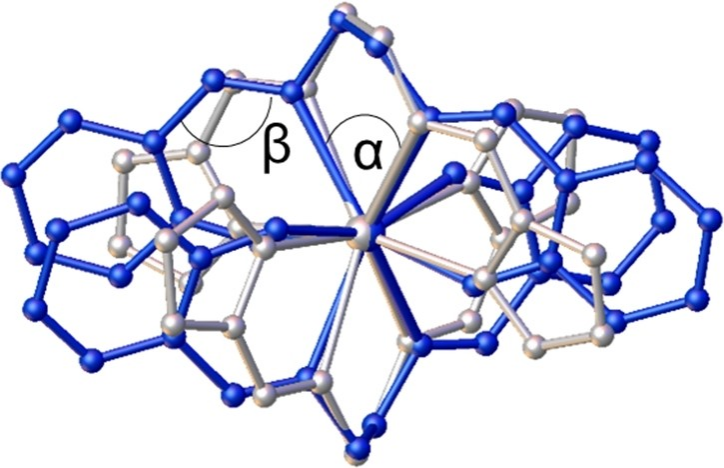
Structural overlay of [U(pyren)_2_] (**2**, silver) and [U(salen)_2_] (**2 a**, blue) complexes (RMSD=0.186 Å).^[7]^

To examine the influence of different donor moieties on the electronic situation in the complexes **1**–**4**, the structurally related and intensely studied *N,N′‐*ethylene‐bis(salicylideneimine) (H_2_salen) based complexes were chosen for comparison. The degree of structural relationship was assessed by an overlay of the crystal structures, exemplary shown for the U compounds (Figure 4). A measure for the similarity of crystal structures is the root mean square deviation (RMSD) of the atomic positions, widely used in protein crystallography.[^24^, ^25^, ^26^] For the two structures a value of 0.186 Å can be found, corresponding to a very good agreement. In Figure 4 it can be seen that the two structures exhibit almost identical coordination environments, however slight deviations arise from differently oriented aromatic rings (pyrrolide vs. phenolate). Especially the bridge and imine region are suitable for comparison, expressed by the almost identical N_imine_–An−N_imine_ angle α, showing a value of 64° and 66° for the [U(pyren)_2_] **2** and [U(salen)_2_] **2 a** complexes, respectively. Nevertheless, the slightly diverging arrangement of the aromatic rings has to be taken into account when discussing structural parameters. The difference is displayed in the C−C−N_imine_ angle *β*, where a deviation of 8° between the structures (118° for **2** and 126° for **2 a**) can be observed. The exchange of the phenolate *O*‐donor versus the pyrrolide *N*‐donor in the five‐membered chelate ring is the obvious explanation. The oxygen is outside the phenyl ring, adding one more atom to the chelate pincer. Thus, the ligand backbone is bent to its limit, as *β* does not vary much with decreasing ionic radius.

**Figure 5:**
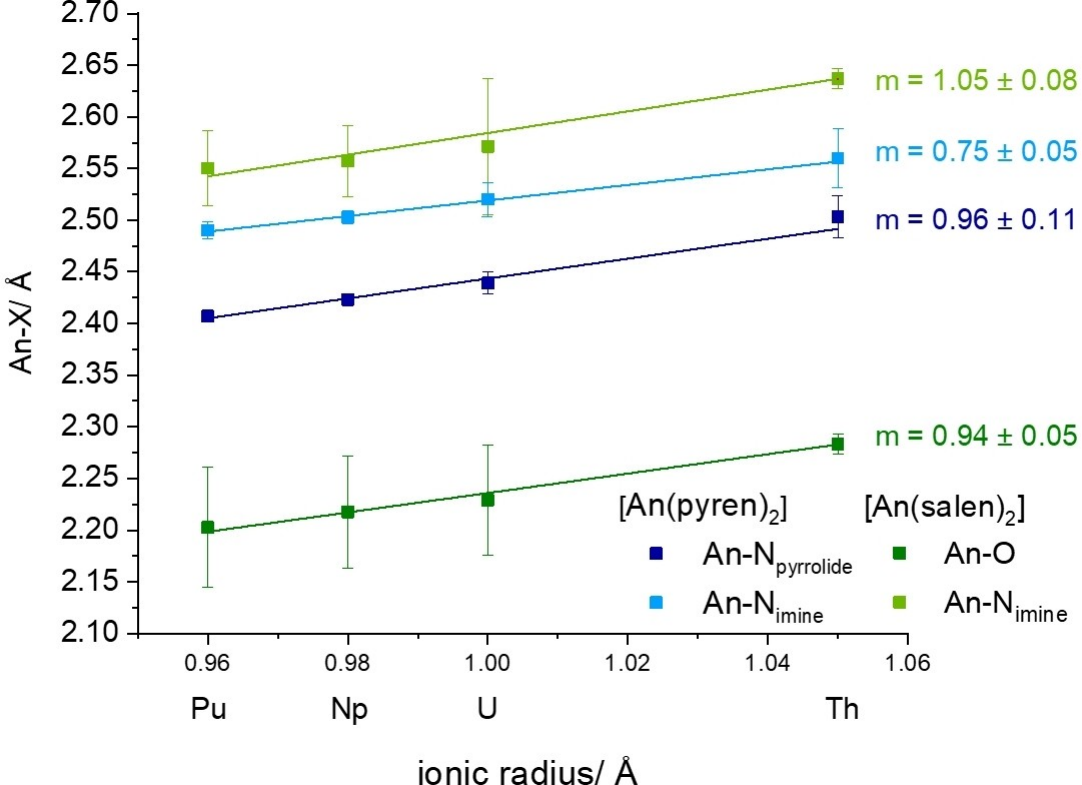
Comparison of An–E (E=O, N) bond lengths in [An(pyren)_2_] and [An(salen)_2_] complexes (An=Th, U−Pu).^[7]^

**Figure 6:**
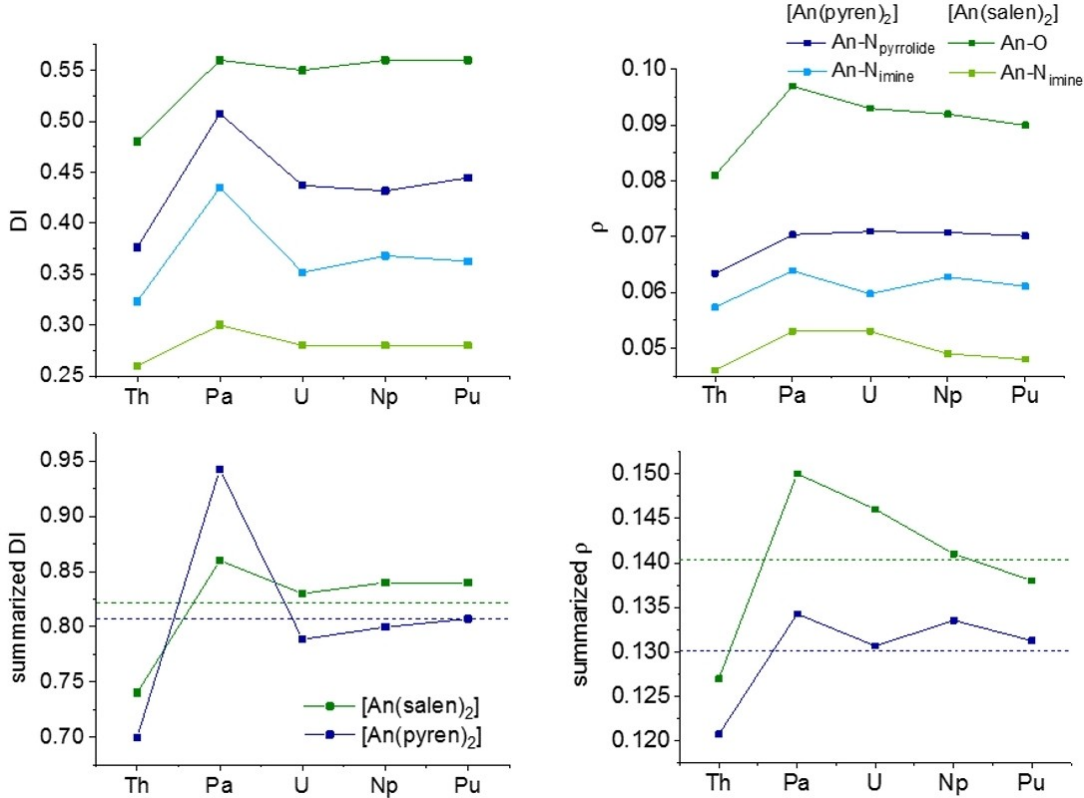
Top: values for the delocalisation index (left) and *ρ* (right) for the An−N_pyrrolide_ and An−N_imine_ bonds in [An(pyren)_2_] complexes (An=Th‐Pu) as well as An−O and An−N_imine_ bonds in [An(salen)_2_] complexes. Bottom: Sum of DI (left) and *ρ* values (right) for An−N_pyrrolide_ and An−N_imine_ bonds in the pyren, An−O and An−N_imine_ in the salen (—) and mean values (‐ ‐ ‐ ‐).

Comparison of the An−E (E=O, N) bond lengths derived from crystal structures clearly shows that the An−O bond is by far the shortest (Figure 5). This can be explained by a combination of a very strong An−O bond and the additional bond between the O atom and the phenyl ring, which brings the donor atom closer to the An. Results from QTAIM and NBO analysis prove the An−O bond to be the strongest, expressed by the highest DI (0.48 for Th and 0.55 for Pa−Pu) and *ρ* values (0.06 for Th and 0.07 for Pa−Pu; Figure 6). This underlines the expected preference of tetravalent actinides for hard oxygen over soft nitrogen. The An−N_imine_ bonds in the pyren complexes, on the other hand, are on average 0.06±0.01 Å shorter than the An−N_imine_ bonds in the salen complexes. Here, quantum chemical calculations prove a greater bond strength and enhanced covalent character for this bond in the pyren complexes. The DI values for An−N_imine_ bonds in the pyren complexes exceed those for the An−N_imine_ in the salen complexes by 0.07 on average. A similar picture can be drawn for the *ρ* values, which are greater by 0.012 on average for the pyren An−N_imine_ bonds compared to the salen An−N_imine_ bonds. The sum of the DI values for both coordinative bonds in the pyren (N_imine_ and N_pyrrolidine_) and the salen (N_imine_ and O) complexes then yield very similar values (0.81 vs. 0.82), with a similar trend for the *ρ* values with 0.13 vs. 0.14 for the pyren and the salen complexes, respectively. This lends further credence to the idea of a relatively rigid ligand cage in which the metal centres summarily interact in a similar fashion for salen and pyren, but with differences along the metal series due to the decreasing ionic radii, and differences for each donor atom depending on hardness.

To assess which ligand forms the more stable complexes we can first compare the DIs and *ρ* values of the two series. The values for the salen complexes mostly exceed the ones of the pyren complexes, indicating the overall bond strength of the two ligands is nearly equal with a slight preference for the salen ligand or a minor preference of the actinides for the salen over the pyren ligand. Experimentally, we can test these findings using a metathesis approach. Upon addition of Li_2_salen to [U(pyren)_2_] only one pyren ligand is displaced by salen resulting in a heteroleptic complex (see the Supporting Information). This is in good agreement with our calculations, showing that the salen ligand is only slightly preferred and thus not capable of replacing both pyren ligands. Once again Pa^IV^ is the exception, where the pyren ligand is significantly favoured in the DIs (salen 0.86 vs. pyren 0.94), which can be explained by the enhanced backbonding effect in the pyren case due to the softer *N*‐donor atoms. Changing the donor moiety from phenolate to pyrrolide results in enhancement of the An−N_imine_ bond strength and covalent character. By choosing the softer N_pyrrolide_ atom as second donor entity, a more even distribution of electron density throughout the molecule is achieved. Although the negative charge is also distributed throughout the conjugated salen molecule within the complexes, the negative charge of the *O*‐donor exceeds that of the *N*‐donor by 0.24 in average.^[7]^ Therefore, the strong *O*‐donor in the salen complexes mainly localises the electron density within the An−O bonds and dominates the bonding environment, leading to a weakening in the An−N_imine_ bond.

## Conclusion

Two Schiff‐base‐type pure *N*‐donor ligand anions [pyren^2−^] can stabilise the actinides Th−Pu in their tetravalent state in overall neutral [An(pyren)_2_] complexes. SC‐XRD analysis revealed the shortest An^IV^−N_pyrrolide/imine_ bonds known in literature so far. In addition, unequal An−N bond lengths, depending on the chemical environment of the donor atom in either the imine or the pyrrolide moiety can be observed. Quantum chemical calculations confirm that the An−N_pyrrolide_ bond is not only shorter, but also stronger, with a greater covalent character. Despite charge delocalisation, an enhanced An−N_pyrrolide_ orbital overlap is responsible for the bond shortening. Furthermore, *f*‐electron participation in bond formation was determined in the form of a backbonding effect. This is expressed in an enhanced An−N bond strength for Pa−Pu compared to Th^IV^. A similar trend was recently found for An−O bonds in a theoretical study including in [An(OC_6_H_5_)_4_] complexes (An=Th‐Pu).^[27]^

Due to the pronounced structural similarity, complexes with the salen ligand were chosen for comparison. It was shown that the An−N_pyrrolide_ and especially the An−N_imine_ bonds in the pyren complexes are not only shorter, but also stronger than the An−N bonds in [An(salen)_2_]. Summed DI and *ρ* values display almost equal bond strength of the salen and pyren ligands, despite the lack of a favoured *O*‐donor in the latter. Thus, changing the second coordinating entity to a less‐dominant donor atom enhances the involvement of the imine nitrogen in An bonding. This can be explained by compensation of a less‐dominant pyrrolide nitrogen as second donor moiety compared to the *O*‐donor in the salen complexes.

Surprisingly, Pa^IV^ forms remarkably strong Pa−N bonds for both kinds of *N*‐donor, leading to an increased complex stability of [Pa(pyren)_2_] over [Pa(salen)_2_]. Hence, the pure *N*‐donor ligand might establish selectivity for Pa^IV^ over the other tetravalent early actinides, which could lead to specified N functionality activation, providing new pathways for reactivity studies, selectivity or catalysis applications. Although no experimental data are available for the Pa complexes, consistency throughout the complex series with Th, U−Pu provides a profound structural basis for quantum chemical calculations. Especially investigations on the bonding situation, for example bond strength, can only be performed by using computational methods. Yet, our findings emphasise that experimental data for Pa remain exceedingly scarce, and experimental studies with this fascinating element are essential for fully understanding the effect of *f*‐electrons on actinide binding and properties. In conclusion, comparing the early actinides Th−Pu as well as the *N*,*O*‐donor salen and the pure *N*‐donor pyren demonstrates the intricate interaction of electronic configuration, Pearson hardness, and covalency in actinide bonding.

## Experimental Section

**Methods**: NMR measurements were performed on a Varian Inova 400 for ^1^H at 400 MHz and ^13^C at 101 MHz resonance frequency in NMR tubes with tightly closed Young‐valve. A Varian AutoX ID head with *z*‐gradient was facilitated to measure ^1^H and ^13^C signals directly at room temperature. Those were correlated for all substances with the help of 2D spectra ^1^H,^1^H COSY, ^1^H,^13^C HSQC and ^1^H,^13^C HMBC spectra. Solid samples were dissolved in 0.6–1.0 mL deuterated THF to reach a concentration of 50–100 mM. Deuterated THF was purchased from Deutero GmbH and stored 24 h over Na/K alloy. Due to solubility issues, the ^13^C NMR spectrum of the Np complex **3** and Pu complex **4** could not be measured.

For the IR data collection in the range of 4000–500 cm^−1^ an Agilent Technologies Cary 630 FTIR spectrometer inside a N_2_‐filled glovebox was facilitated, and a resolution of 1 cm^−1^ was reached. For the measurement, the powdered sample was pressed onto the ATR crystal (diamond).

Single crystal X‐ray diffraction analysis was performed on a Bruker D8 Venture diffractometer using a micro focus Mo_Kα_ source (*λ*=0.71073 Å) and a Photon 100 CMOS detector. To avoid crystal damage through oxygen and moisture, the samples were placed into a continuous N_2_ flow provided by a Oxford Cryosteam system and measured at 100 K. Suitable crystals were selected by using an optical microscope and mounted on a MicroMount (MiTeGen, USA). Data were collected through generic *ϕ* and *ω* scans, and integrated with Bruker APEX 3 software with SAINT package. Empirical absorption correction was performed through the multi scan method (SADABS). Structure solution was conducted by full‐matrix least‐squares method on *F* ^2^ with the Bruker SHELXTL package. All non‐hydrogen atoms were anisotropically refined, placed at calculated positions and allowed to ride on their parent atoms. The thorium complex **1** was refined as a two‐component inversion twin with a flack parameter of 0.26(3).

For the elemental analysis of C, N and H a vario MICRO cube (Elementar) was used. Measurements were conducted under He flow. Np and Pu containing samples were not measured due to radiological safety reasons.

The melting point was determined by using a Stuart Scientific SMP 3 device. The powdered sample was filled into a glass capillary and heated with rate of 5 °C/ min. Shortly before reaching the melting point, the rate was decreased to 1 °C/min. The process was stopped the moment all of the substance was liquified.

**Quantum chemical calculations**. All structures were optimised by using the Turbomole package version 7.3.1. All optimisations were performed at the DFT level using the PBE0 functional. This functional has proven to be very reliable for actinide complexes in previous studies. Scalar relativistic effects were included via a small core ECP for the actinides with a corresponding basis set. Dispersion effects were considered by the Grimme approximation.

In a first step the structures were optimised with the def‐SVP basis set for all atoms. These pre‐optimised structures were then optimised with a def‐TZVPP basis set. Vibrational frequencies were then calculated numerically (using the NumForce script) and checked to ensure a true minimum on the potential hypersurface. Based on these optimised structures an NBO analysis was performed with the NBO code implemented in Turbomole. The structures were then used to perform single‐point all‐electron calculations with Orca version 4.2.1 using the SARC‐DKH‐TZVPP basis set for the actinides and the DKH‐def2‐TZVPP basis set for all other elements. Scalar relativistic effects were included by using the Douglas–Kroll–Hess approximation. From the results of this calculation, input files for the QTAIM analysis were generated with the molden2aim code version 5.0.2. The QTAIM analysis was then performed with the AIMALL code version 19.10.12. Density‐difference plots were generated by calculating the electron density of the complex, the ligand cage and the naked actinide ion at the optimised structure of the complex. This was done with Orca. The actual difference was generated with the MultiWFN code version 3.7. The resulting difference plots were visualised with VMD version 1.9.4a43. To test the validity of the DFT calculation for the open‐shell actinide systems NEVPT2 calculations were performed using Orca. The active space was chosen as the *f*‐shell of the actinide ion. To ensure the correct active space a DFT calculation was performed on the complexes in which all *f*‐electrons were removed. The MO's of this calculation were checked to ensure pure *f*‐orbitals in the active space. Then the NEVPT2 calculations were performed using the previous DFT orbitals as starting guess.

**Chemicals and procedures**: ***CAUTION***! Thorium, uranium, neptunium and plutonium consist of radioactive nuclides including long‐lived α‐emitters (^232^Th: *t*_1/2_=1.41×10^10^ years, ^235^U: 7.04×10^8^ years, ^238^U: 4.47×10^9^ years, ^237^Np: 2.14×10^6^ years, ^242^Pu: 373×10^3^ years). For safe handling, special precautions and equipment are necessary. Therefore, all the experiments were conducted in the controlled laboratory at the Institute of Resource Ecology, Helmholtz‐Zentrum Dresden–Rossendorf.

All chemicals were used a received. Solvents were purified and dried through a two‐column solvent purification system by MBraun (MBSPS 5), equipped with activated Al_2_O_3_ and stored over 3 Å molecular sieve afterwards. Metal precursors UCl_4_, ThCl_4_(dme)_2_, NpCl_4_(dme)_2_, PuCl_4_(dme)_2_ as well as the ligand were synthesised according to literature.[^28^, ^29^, ^30^, ^31^]

Complex synthesis were performed in N_2_‐filled gloveboxes (MBraun, H_2_O <0.5 ppm, O_2_ <0.5 ppm) and by standard Schlenk techniques to exclude air and moisture.

**H_2_pyren (H_2_L)**: A solution of 1 g (0.011 mol, 2 equiv) pyrrol‐2‐carboxaldehyde in approximately 15 mL abs. ethanol and 0.35 mL (0.005 mol, 1 equiv) ethylendiamine were stirred at room temperature for 10 min. Then, 5 drops of formic acid were added, and after another 10 min of stirring the product started to precipitate as a white powder. After one day, the product was separated through filtration and washed four times with small amounts of cold ethanol, followed by drying in vacuum. Yield: 0.638 g (60 m.p.: decomposition 180 °C. ^1^H NMR: (400 MHz, [D_8_]THF): *δ*=0.69 (s, 1H, N*H*), 8.01 (s, 1H, N_I_C*H*), 6.78 (m, 1H, C*H*), 6.32 (dd, *J*=3.6, 1.5, 1H, C*H*), 6.06 (m, 1H, N_P_C*H*), 3.71 ppm (s, 2H, C*H_2_*).^13^C NMR: (101 MHz, [D_8_]THF): *δ*=152.9 (N_I_ *C*H), 131.7 (N_P_C_q_), 122.2 (*C*H), 114.2 (*C*H), 109.5 (N_P_ *C*H), 63.2 ppm (*C*H_2_). IR (ATR): ν [cm^−1^] 723 (vs), 751 (m), 787 (w), 823 (m), 882 (w), 972 (w), 1015 (s), 1036 (w), 1048 (m), 1102 (m), 1130 (s), 1202 (w), 1248 (w), 1288 (m), 1315 (m), 1351 (vw), 1418 (s), 1445 (vw), 1475 (w), 1552 (vw), 1633 (s), 2861 (m), 2943 (m), 3153 (m), 2500–3300 (very broad). UV/Vis (THF) [nm]: *λ*_max_ 351.5.EA: calcd (%) for C_12_H_14_N_4_: C 67.1, H 6.7, N 26.0; found: C 67.3, H 6.6, N 26.2.

**[Th^IV^(pyren)_2_]⋅2 MeCN**: H_2_ **L** (25.2 mg, 0.12 mmol, 2 equiv) was deprotonated with excess of lithium diisopropylamide (LDA) (26.4 mg, 0.25 mmol, 4 equiv) in THF through stirring at room temperature for 30 min. The initially clear solution changed to cloudy white, as the ligand lithium salt forms. After the addition of ThCl_4_(dme)_2_ (32.2 mg, 0.06 mmol, 1 equiv) in 1 mL THF, the colour changed to light yellow, and the solution turned clear again. After one day, the volatile components were removed under reduced pressure, resulting in a white solid. The crude product was recrystallised from hot acetonitrile to yield white to pale yellow crystals, suitable for SC‐XRD. yield: 33.5 mg (85 %); ^1^H NMR (400 MHz, [D_8_]THF): *δ*=8.11 (s, 1H, N_I_C*H*), 6.83 (m, 1H, N_P_C*H*), 6.27 (dd, *J*=3.3, 1.1, 1H, N_P_C*H*), 5.69 (dd, *J*=3.4, 1.8, 1H, N_P_C*H*), 4.04 ppm (s, 2H, C*H*_2_). ^13^C NMR (101 MHz, [D_8_]THF): *δ*=160.6 (N_I_ *C*H) 139.0 (N_P_ *C_q_*), 136.4 (N_P_ *C*H), 118.0 (N_P_ *C*H), 109.3 (N_P_ *C*H), 58.5 ppm (*C*H_2_). IR [cm^−1^]: 686 (vw), 737 (vs), 778 (w), 794 (w), 845 (w), 891 (s), 914 (vw), 943 (m), 972 (s), 1028 (vs), 1085 (w), 1178 (w), 1202 (w), 1238 (w), 1252 (w), 1301 (s), 1326 (m), 1348 (w), 1395 (m), 1431 (m), 1460 (vw), 1506 (w), 1582 (vs), 2873 (w, br t), 3083 (w). UV/Vis (MeCN) [nm]: 289, 342. *λ*_max_ 300, 350. EA calcd (%) for C_24_H_24_N_8_Th: C 43.9, H 3.7, N 17.1; found: C 43.6, H 3.2, N 16.8.

**[U^IV^(pyren)_2_]⋅2 MeCN**: A mixture of H_2_ **L** (49.9 mg, 0.23 mmol, 2 equiv) and LDA (51.0 mg, 0.48 mmol, 4 equiv) in 2 mL THF were stirred at room temperature for approximately 30 min. Upon addition of UCl_4_ (44.0 mg, 0.12 mmol, 1 equiv) in 1 mL THF an immediate colour change to orange brown occurred, and the product started to precipitate after 15 min. Stirring for one day at room temperature yielded an orange microcrystalline solid that was separated from the reaction mixture through centrifugation and washed with small amounts of THF. Recrystallisation from hot acetonitrile gave crystals suitable for SC‐XRD. Yield: 69.9 mg (91 %). ^1^H NMR (400 MHz, [D_8_]THF): *δ*=18.34 (d, *J*=4.0, 1H, N_P_C*H*), 16.81 (s, 1H, N_I_C*H*), 16.18 (d, *J*=4.0, 1H, N_P_C*H*), 7.01 (s, 1H, N_P_C*H*), −29.71 ppm (s, 1H, C*H_2_*). ^13^C NMR (101 MHz, [D_8_]THF): *δ*=169.6 (N_P_ *C*H), 160.2 (N_P_ *C*H), 153.0 (N_P_ *C*H), 77.2 (N_P_ *C_q_*), 35.9 (N_I_ *C*H), −29.3 ppm (*C*H_2_). IR [cm^−1^]: 684 (w), 741 (vs), 776 (w), 792 (w), 840 (w), 889 (m), 940 (m), 973 (s), 1030 (vs), 1045 (s), 1078 (w), 1086 (m), 1174 (w), 1198 (m), 1235 (w), 1249 (m), 1300 (s), 1326 (s), 1345 (m), 1396 (s), 1427 (s), 1443 (m), 1509 (w), 1579 (vs), 2642 (vw), 2852 (w), 2913 (w), 2924 (w), 3095 (w). UV/Vis (MeCN) [nm]: *λ*_max_ 300–380 (br), 465. EA calcd (%) for C_24_H_24_N_8_U: C 43.5, H 3.7, N 16.9; found: C 42.8, H 3.8, N 16.2.

[**Np^IV^(pyren)_2_]⋅2 MeCN**: The deprotonation of H_2_ **L** (12.7 mg, 0.06 mmol, 2 equiv) through LDA (14.1 mg, 0.13 mmol, 4 equiv) was conducted in 1 mL THF through stirring at room temperature for 30 min. The addition of NpCl_4_(dme)_2_ (16.6 mg, 0.03 mmol, 1 equiv) in 1 mL THF resulted in an instant colour change from light yellow to intense orange‐red and shortly thereafter an orange solid precipitated. After stirring over night at room temperature, the mixture was centrifuged; the solid washed with two portions of THF each and dried. The volatile components of the supernatant were removed under reduced pressure and combined crops were recrystallised from hot acetonitrile. Orange platelets suitable for SC‐XRD were obtained. yield: 16.0 mg (81 %). ^1^H NMR (400 MHz, [D_8_]THF): *δ*=20.32 (s, 1H, N_I_C*H*), 17.32 (m, 1H, N_P_C*H*), 13.55 (s, 1H, N_P_C*H*), 7.88 (s, 1H, N_P_C*H*), −17.52 ppm (s, 2H, C*H_2_*). IR [cm^−1^]: 684 (vw), 741 (s), 757 (vw), 777 (w), 794 (w), 841 (w), 889 (w), 942 (m), 975 (s), 1031 (s), 1044 (s), 1079 (m), 1087 (m), 1175 (w), 1199 (m), 1235 (w), 1249 (m), 1300 (s), 1328 (s), 1346 (m), 1396 (s), 1428 (s), 1444 (w), 1508 (w), 1580 (s, shoulder 1592), multiple very weak bands 2286–2637, 2852 (w), 2915 (vw), 2927 (vw), 3100 (vw). UV/Vis/NIR (MeCN) [nm]: 286, 357, 427 (shoulder), 735, 871.

**[Pu^IV^(pyren)_2_]⋅2 MeCN**: A mixture of H_2_ **L** (12.5 mg (0.06 mmol, 2 equiv) and excess of LDA (13.1 mg, 0.12 mmol, 4 equiv) in approximately 0.5 mL THF was stirred at room temperature for 30 min. The addition of PuCl_4_(dme)_2_ (15.9 mg, 0.03 mmol, 1 equiv) in 0.5 mL THF caused the mixture to change the initial light yellow colour to dark brown. After 1.5 d, a dark, metallic precipitate was separated through centrifugation from a clear brown supernatant. The crude product was recrystallised from hot acetonitrile to yield black‐brown block shaped crystals suitable for SC‐XRD. yield: 13.9 mg (70 %). ^1^H NMR (400 MHz, [D_8_]THF): *δ*=15.65 (s, 1H, N_I_C*H*), 10.18 (dd, 1H, N_P_C*H*), 7.65 (q, 1H, N_P_C*H*), 7.49 (s, 1H, N_P_C*H*), 3.23 ppm (s, 2H, C*H*_2_). IR [cm^−1^]: 684 (vw), 741 (s), 757 (vw), 777 (w), 794 (w), 841 (w), 889 (w), 942 (m), 975 (s), 1031 (s), 1044 (s), 1079 (m), 1087 (m), 1175 (w), 1199 (m), 1235 (w), 1249 (m), 1300 (s), 1328 (s), 1346 (m), 1396 (s), 1428 (s), 1444 (w), 1508 (w), 1580 (s, shoulder 1592), multiple very weak bands 2286–2637, 2852 (w), 2915 (vw), 2927 (vw), 3100 (vw). UV/Vis (MeCN) [nm]: 385–375 (br), 817, 850, 1066, 1112, 1166.

Deposition numbers 2101417 (for **1**), 2101418 (for **2**), 2101419 (for **3**), and 2101420 (for **4**) contain the supplementary crystallographic data for this paper. These data are provided free of charge by the joint Cambridge Crystallographic Data Centre and Fachinformationszentrum Karlsruhe Access Structures service.

## Conflict of interest

The authors declare no conflict of interest.

## Supporting information

As a service to our authors and readers, this journal provides supporting information supplied by the authors. Such materials are peer reviewed and may be re‐organized for online delivery, but are not copy‐edited or typeset. Technical support issues arising from supporting information (other than missing files) should be addressed to the authors.

Supporting Information
