# Supplementary material for: How 5 f Electron Polarisability Drives Covalency and Selectivity in Actinide N‐Donor Complexes
Source: Chemistry. 2021 Dec 2;27(72):18058–65. doi: 10.1002/chem.202102849 (PMC9299701; doi:10.1002/chem.202102849)
Supplement: Supplementary file 1 — Supporting Information [file CHEM-27-18058-s001.pdf]

Chemistry–A European Journal

Supporting Information

How 5 *f* Electron Polarisability Drives Covalency and Selectivity in Actinide *N*-Donor Complexes

Luisa Köhler, Michael Patzschke, Moritz Schmidt, Thorsten Stumpf, and Juliane März*

Table of Contents

1. NMR Data 2
2. UV-VIS NIR Data 6
3. Crystallographic Data 7
4. Structural Data and Geometrical Analysis 10
5. IR Data 12
6. Computational Details 13

1. NMR Data

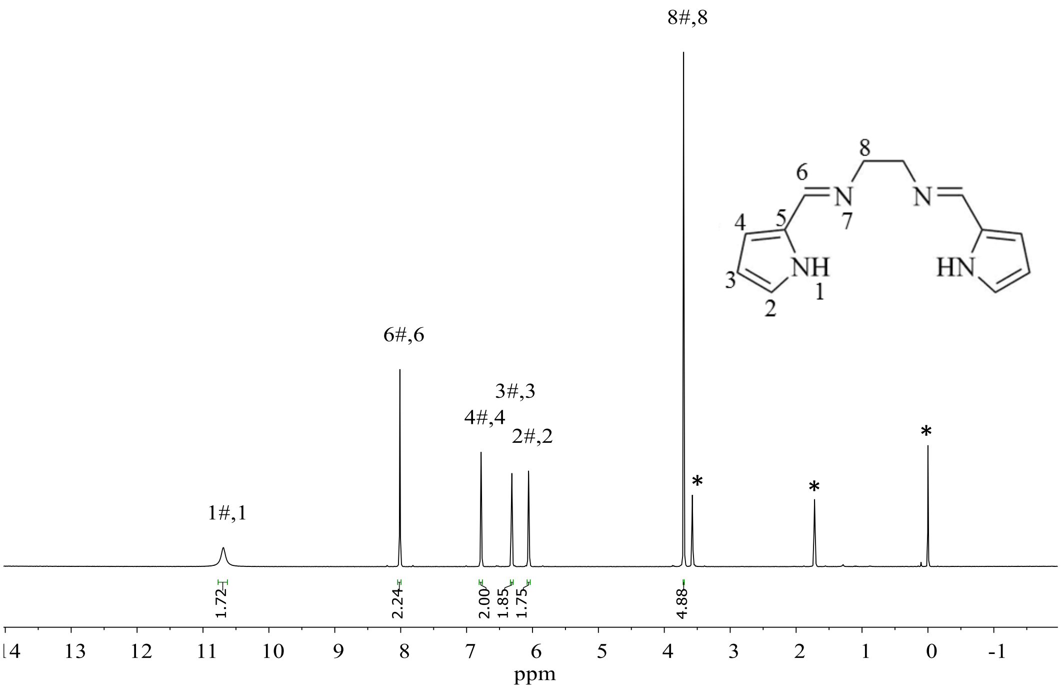

Figure 1 ^{1}H NMR spectrum of the ligand H_2**L** (H_2pyren) in THF-d_8 measured at room temperature. Solvent and TMS (tetramethylsilane) signals are marked with an asterisk.

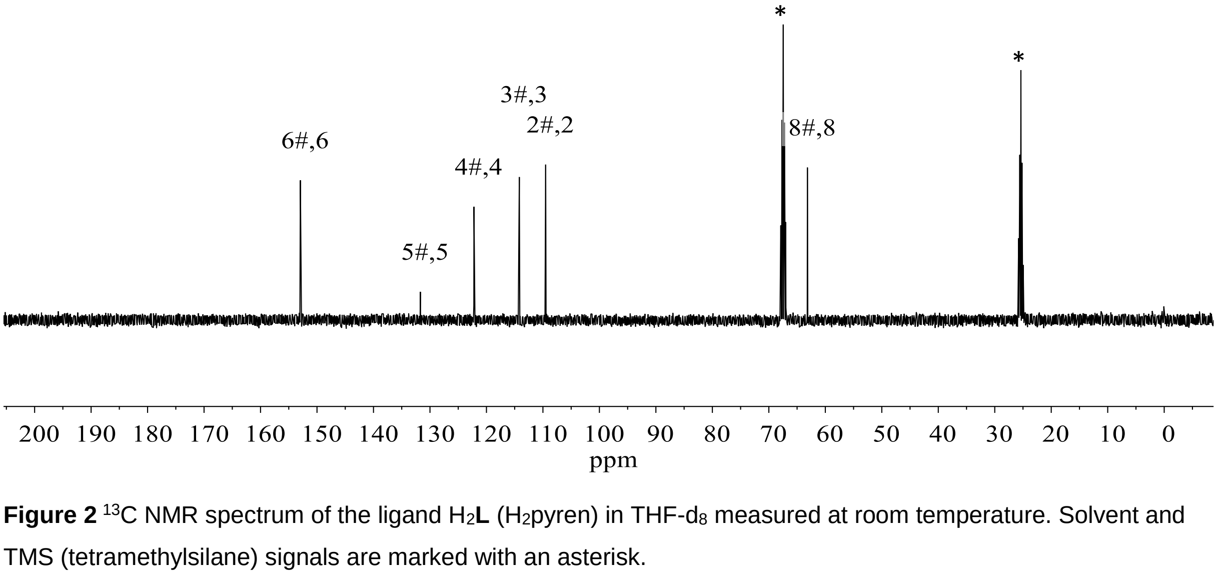

Figure 2 ^{13}C NMR spectrum of the ligand H_2**L** (H_2pyren) in THF-d_8 measured at room temperature. Solvent and TMS (tetramethylsilane) signals are marked with an asterisk.

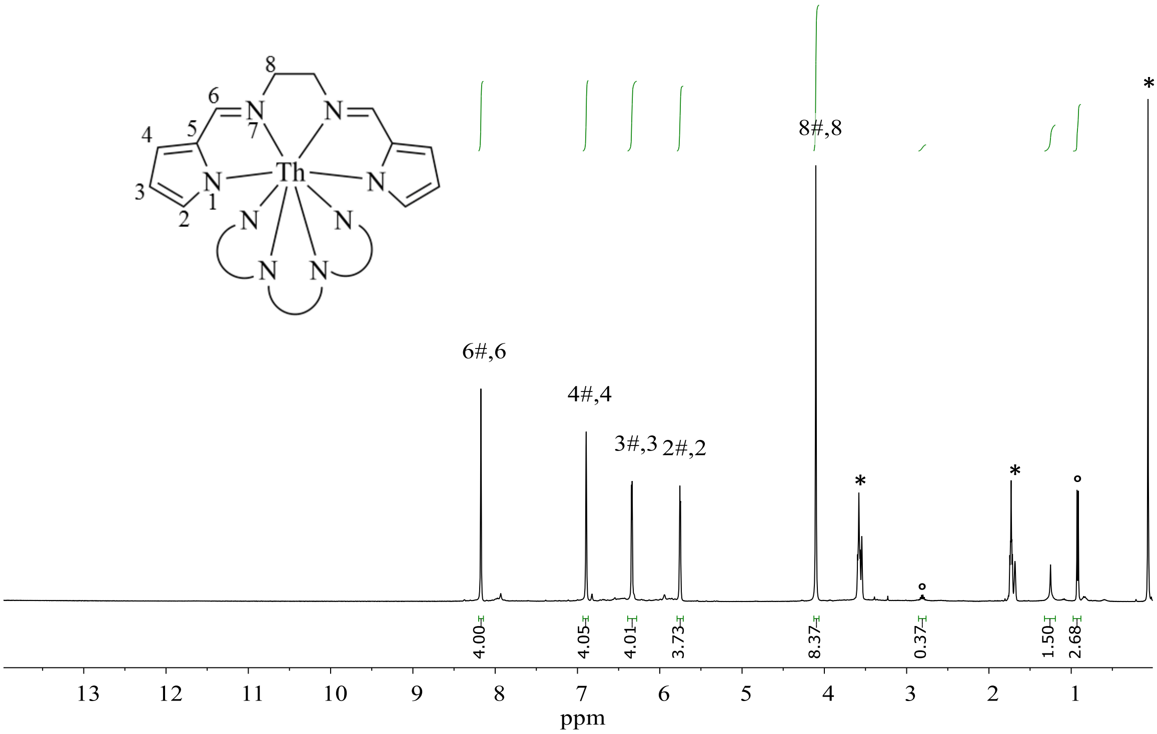

Figure 3 ^{1}H NMR spectrum of compound **1** [Th(pyren)$_2$] in THF-d$_8$ measured at room temperature. Solvent and TMS (tetramethylsilane) signals are marked with an asterisk; traces of lithium diisopropylamide (LDA) are marked with a circle.

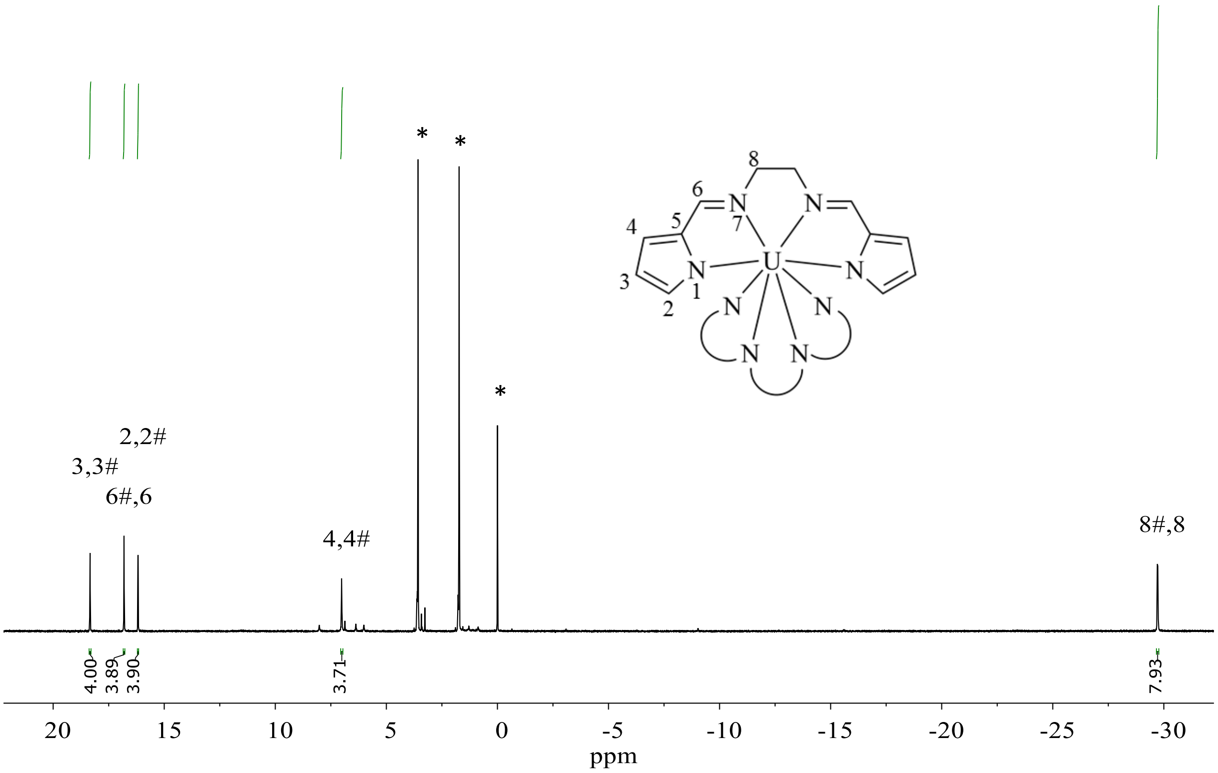

Figure 4 ^{1}H NMR spectrum of compound **2** [U(pyren)$_2$] in THF-d$_8$ measured at room temperature. Solvent and TMS (tetramethylsilane) signals are marked with an asterisk.

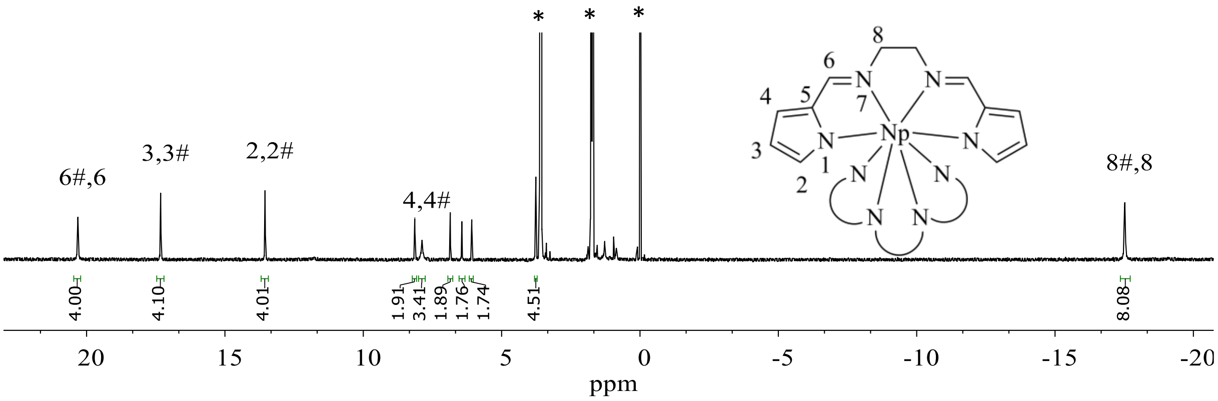

Figure 5 ^{1}H NMR spectrum of compound **3** [Np(pyren)$_2$] in THF-d$_8$ measured at room temperature. Solvent and TMS (tetramethylsilane) signals are marked with an asterisk, signals at 4 ppm and 6-8 ppm correspond to free ligand.

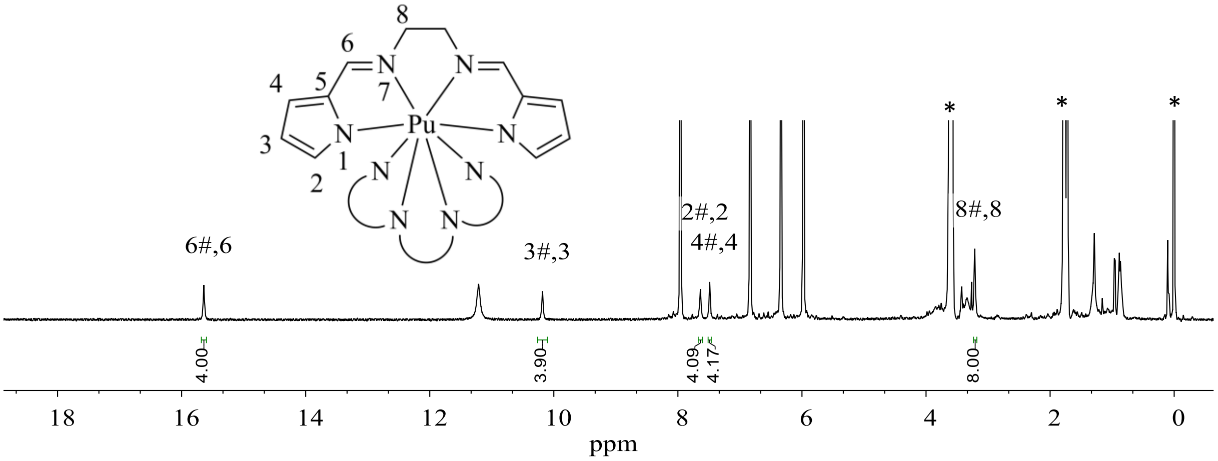

Figure 6 ^{1}H NMR spectrum of compound **4** [Pu(pyren)$_2$] in THF-d$_8$ measured at room temperature. Solvent and TMS (tetramethylsilane) signals are marked with an asterisk; signals at 4 ppm, 6-8 ppm and 11 ppm correspond to free ligand.

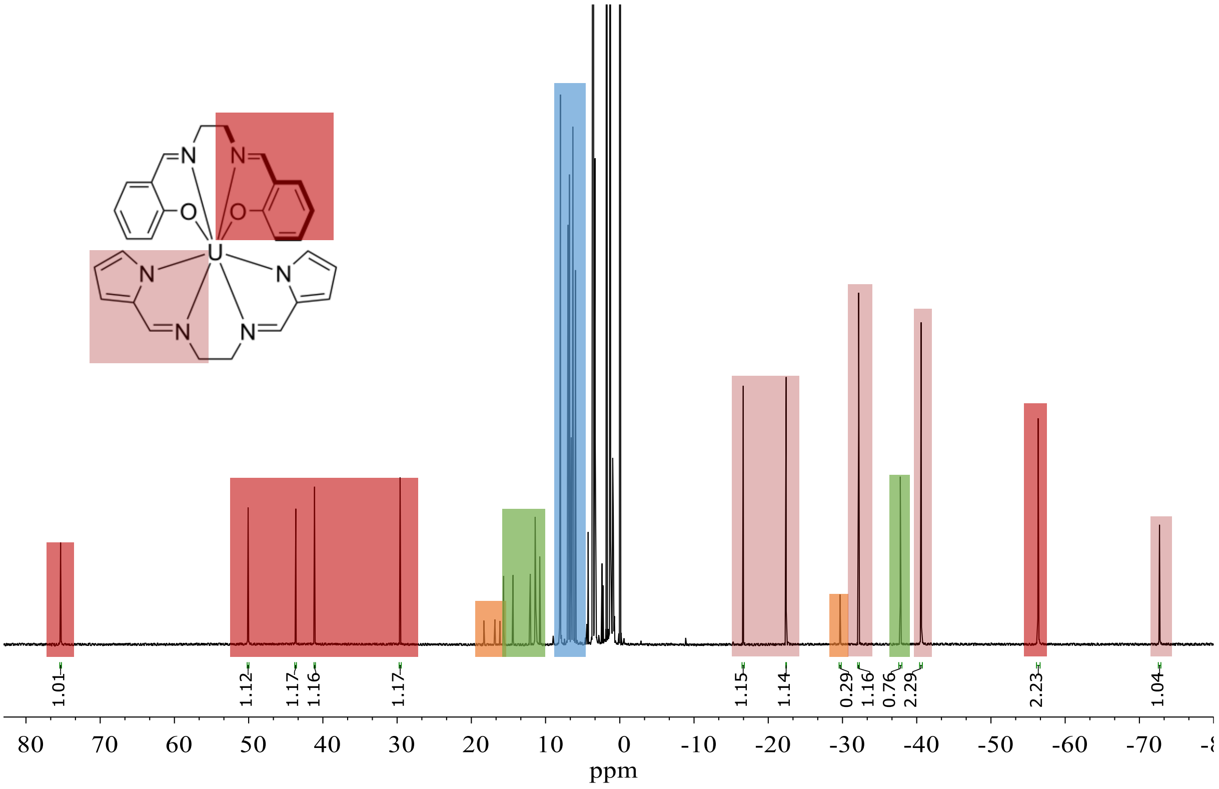

Figure 7 ^{1}H NMR spectrum of the reaction between [U(pyren)$_2$] and Li_2(salen) in THF-d_8. Besides the signals from [U(pyren)$_2$] (orange), [U(salen)$_2$] (green) and free ligands Li_2pyren and Li_2(salen) (blue), the major species is the heteroleptic complex [U(salen)(pyren)] (red).

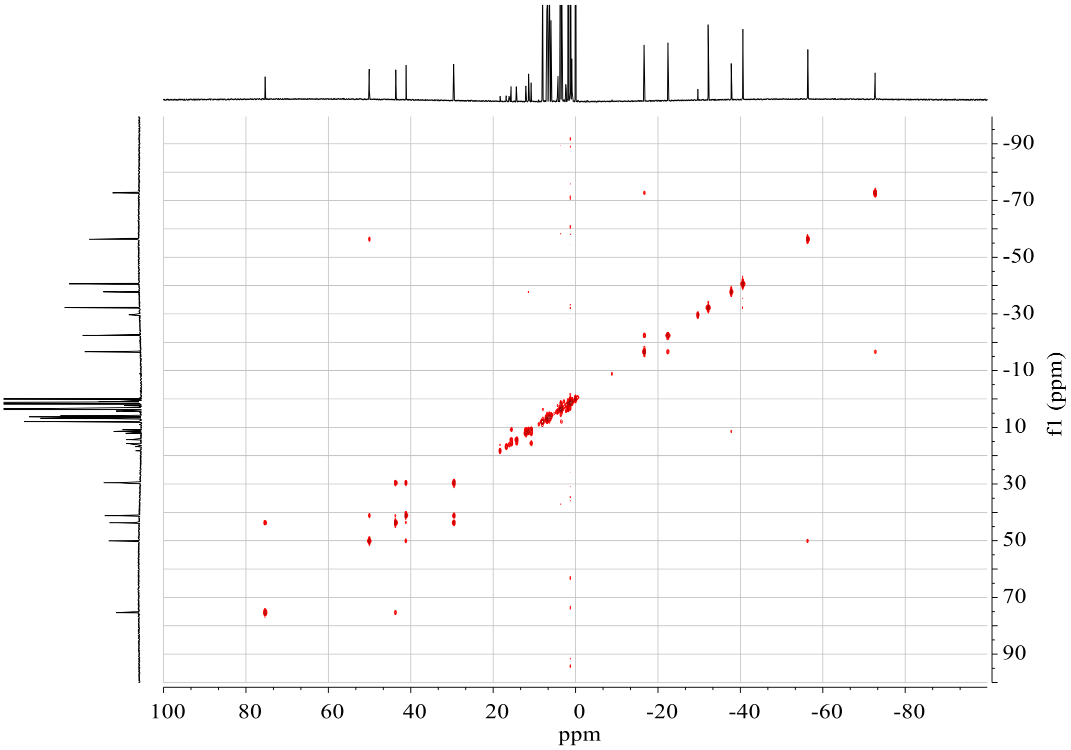

Figure 8 ^{1}H-^{1}H COSY spectrum of the reaction between [U(pyren)$_2$] and Li_2(salen) in THF-d_8.

2. UV-VIS NIR Data

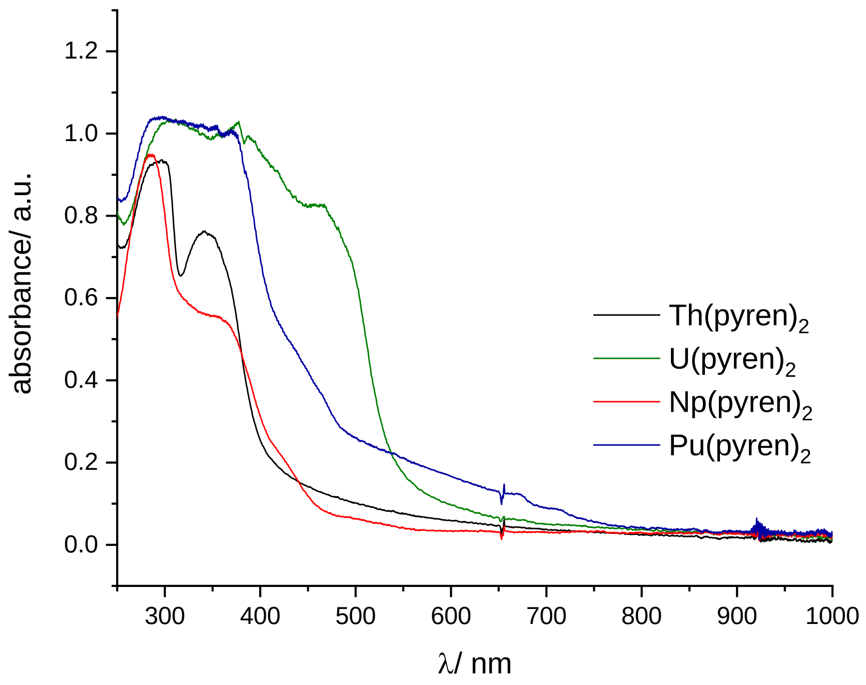

Figure 9 UV-VIS spectra of [An(pyren)2] (An=Th, U-Pu) complexes **1**-**4** at 100µM concentration in acetonitrile.

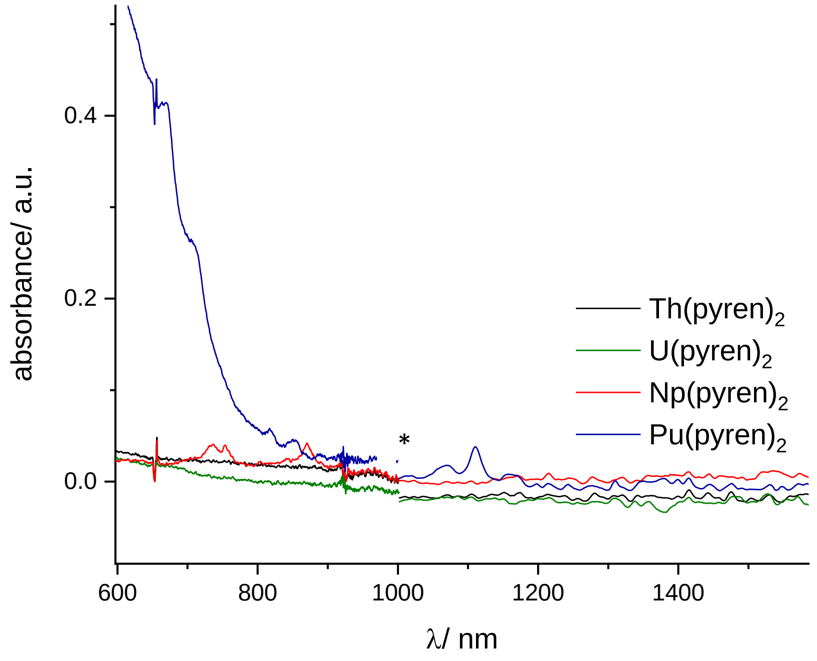

Figure 10 VIS-NIR spectra of [An(pyren)2] (An=Th, U-Pu) complexes **1**-**4** at 0.3 mM concentration in acetonitrile. The asterisk marks change of measurement setting (detector and optical cables).

3. Crystallographic Data

Table 1 SC-XRD data for the reported complexes **1**-**4**.

	**1**	**2**	**3**	**4**
**Molecular formula**	$C_{24}H_{24}N_8Th{\cdot}2\ C_2H_3N$	$C_{24}H_{24}N_8U{\cdot}2\ C_2H_3N$	$C_{24}H_{24}N_8Np{\cdot}2\ C_2H_3N$	$C_{24}H_{24}N_8Pu{\cdot}2\ C_2H_3N$
**CCDC**	2101417	2101418	2101419	2101420
**M (g/mol)**	738.7	741.6	743.6	748.6
**Crystal System**	orthorhombic	orthorhombic	orthorhombic	orthorhombic
**Space Group**	$Pca2_1$	$Pca2_1$	$Pca2_1$	$Pca2_1$
***a* (Å)**	28.168 (4)	27.789 (2)	27.6853(10)	27.5626 (11)
***b* (Å)**	11.1381 (15)	11.1670 (9)	11.1906 (4)	11.2275 (5)
***c* (Å)**	9.1453 (13)	9.0604 (6)	9.0141 (3)	8.9853 (4)
***α* (°)**	90	90	90	90
***β* (°)**	90	90	90	90
***γ* (°)**	90	90	90	90
***V* (Å^3)**	2869.2 (7)	2811.6 (4)	2792.7 (2)	2780.6 (2)
***T* (K)**	100	100	100	100
Z	4	4	4	4
**$\rho_{calc..}$ (mg/m^3)**	1.710	1.752	1.769	1.788
**abs. coeff. (mm^{-1})**	5.233	5.81	3.758	2.408
**Θ_{max} (°)**	23.281	26.8	26.0	25.8
**R [$I > 2\sigma(I)$]**	0.0411	0.0255	0.020	0.025
**Flack parameter**	0.26(3)	0.033(11)	0.014 (10)	−0.002(11)
**wR_2(int)**	0.0915	0.651	0.035	0.038
w* scheme[a] *d*; *e	0.0210; −27.3487	0.0312; -	0.0091; 0.5395	0.0097; 0.3020
**Data/Param.**	5054/ 331	5731/ 355	4919/354	5900/ 336
**Res. Density (Å^{-3}) Max; Min**	2.787; −1.423	1.959; −1.442	0.351; −0.426	1.220; −0.901
**R_{int}**	0.0387	0.0641	0.0419	0.0416
**GooF**	1.10	1.05	1.01	1.02

[a] $w=1/[\sigma^2 I_o^2+(dP)^2+eP]$, where $P=(I_o^2+2I^2)/3$

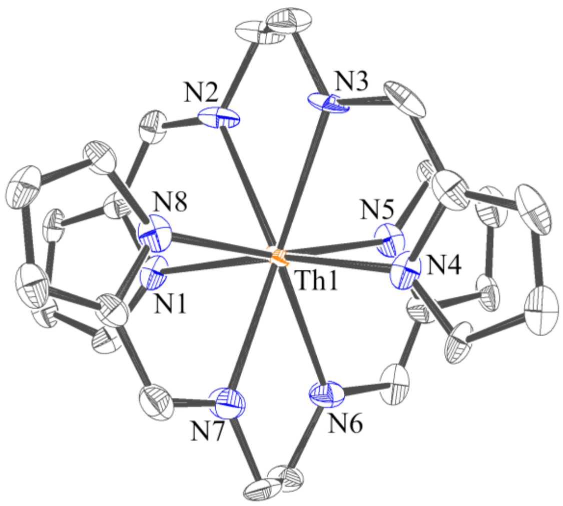

Figure 11 ORTEP plot of the molecular structure of the [Th(pyren)$_2$] complex (**1**). Protons and solvent molecules are omitted for clarity. Ellipsoids are drawn at 30% probability level. Color code: carbon – C, grey, nitrogen – N, blue, thorium – Th, orange.

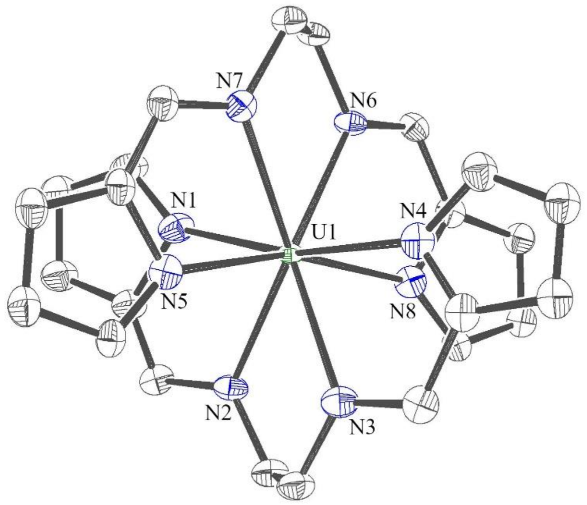

Figure 12 ORTEP plot of the molecular structure of the [U(pyren)$_2$] (**2**) complex. Protons and solvent molecules are omitted for clarity. Ellipsoids are drawn at 50% probability level. Color code: carbon – C, grey, nitrogen – N, blue, uranium – U, green.

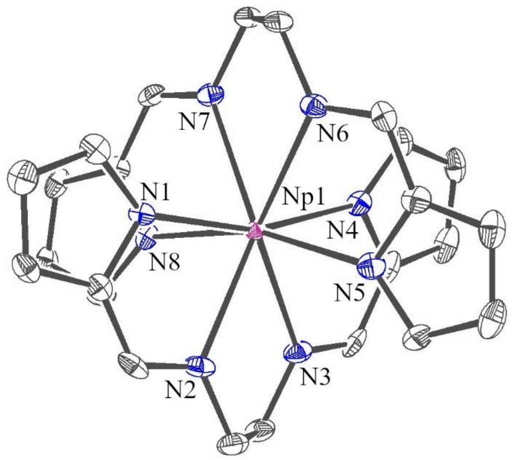

Figure 13 ORTEP plot of the molecular structure of the [Np(pyren)$_2$] (**3**) complex. Protons and solvent molecules are omitted for clarity. Ellipsoids are drawn at 50% probability level. Color code: carbon – C, grey, nitrogen – N, blue, neptunium – Np, magenta.

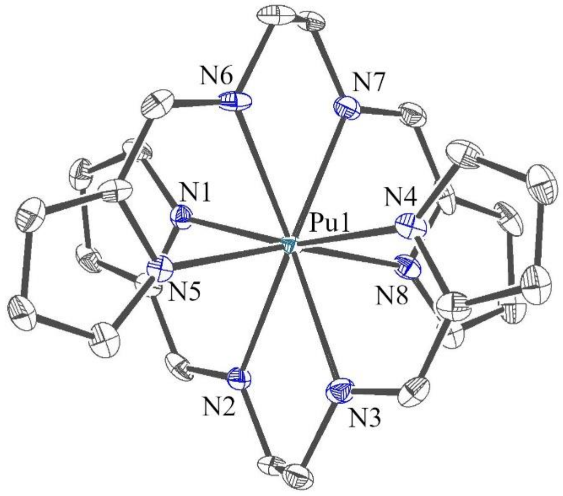

Figure 14 ORTEP plot of the molecular structure of the [Pu(pyren)$_2$] (**4**) complex. Protons and solvent molecules are omitted for clarity. Ellipsoids are drawn at 50% probability level. Color code: carbon – C, grey, nitrogen – N, blue, plutonium – Pu, teal.

4. Structural Data and Geometrical Analysis

Table 2 Relevant bond lengths (Å) and angles (°) in the reported [An(pyren)$_2$] complexes **1-4**.

		**1**	**2**	**3**	**4**
**An-N$_{pyrrol}$**	**-N1**	2.532 (11)	2.439 (4)	2.423 (4)	2.406 (5)
	**-N4**	2.494 (12)	2.429 (4)	2.432 (5)	2.399 (5)
	**-N5**	2.508 (12)	2.441 (4)	2.420 (5)	2.417 (5)
	**-N8**	2.476 (12)	2.447 (4)	2.417 (5)	2.407 (5)
**An-N$_{imine}$**	**-N2**	2.601 (14)	2.514 (4)	2.511 (5)	2.494 (5)
	**-N3**	2.562 (11)	2.516 (5)	2.505 (5)	2.478 (5)
	**-N6**	2.555 (12)	2.529 (5)	2.500 (5)	2.490 (5)
	**-N7**	2.521 (14)	2.522 (4)	2.494 (4)	2.499 (5)
**angle α**	**N2-An-N3**	62.7 (4)	62.9 (2)	63.3 (2)	63.5 (2)
	**N6-An-N7**	61.2 (4)	63.4 (1)	63.5 (2)	63.6 (2)
**angle β**	**C4-C5-N2**	120.0 (14)	119.7 (4)	120.8 (6)	120.6 (6)
	**C9-C8-N3**	121.6 (15)	119.6 (5)	120.3 (6)	119.2 (6)
	**C16-C17-N6**	119.9 (15)	119.7 (5)	120.0 (6)	120.3 (6)
	**C21-C20-N7**	121.2 (15)	119.6 (5)	119.7 (5)	120.9 (6)
**torsion angle bridge**	**N6-C18-C19-N7**	42.4 (18)	41.5 (6)	40.3 (7)	40.0 (6)*
	**N2-C6-C7-N3**	43.6 (20)	40.7 (5)	40.2 (6)	39.8 (9)*

* absolute value

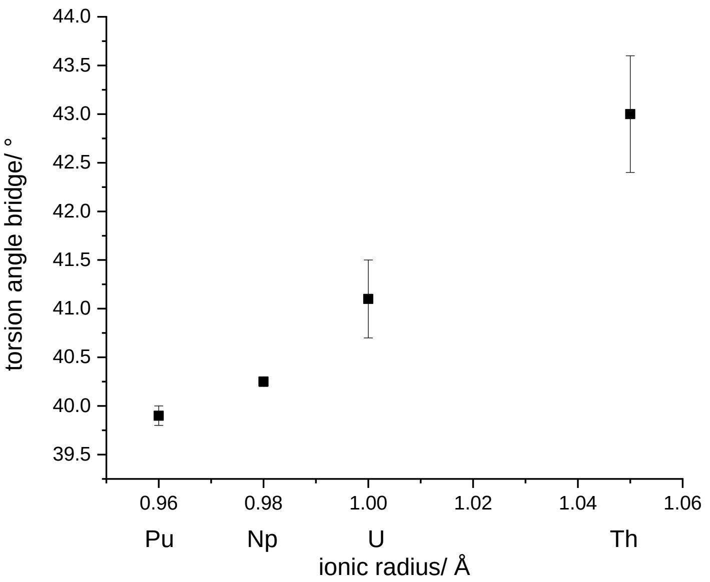

Figure 15 Mean value of bridge torsion angles plotted against the ionic radii of the metal centres.

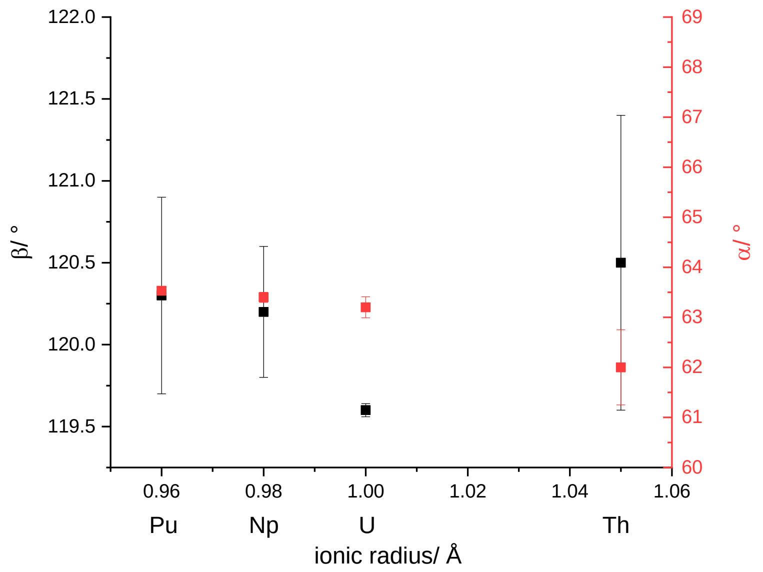

Figure 16 Plot of the N-An-N angle α (red) and the C-C-N angle β (black) against the ionic radii of the metal centres.

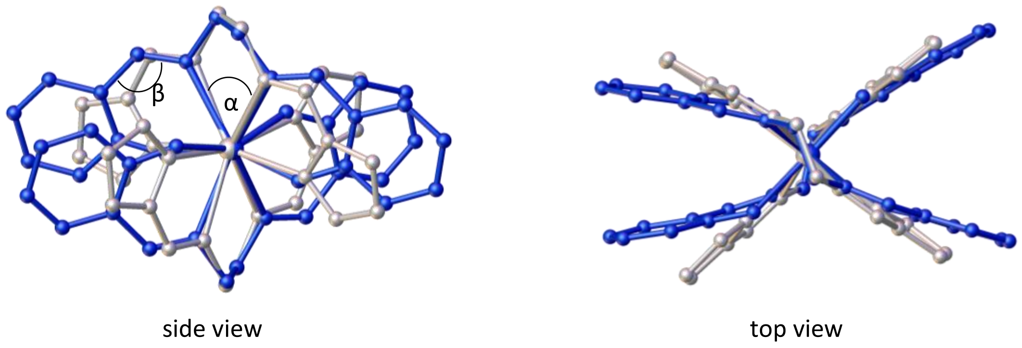

Figure 17 Structural overlay of [U(pyren)$_2$] (silver) and [U(salen)$_2$] (blue) complexes (root mean square deviation (RMSD) 0.186 Å).[5]

5. IR Data

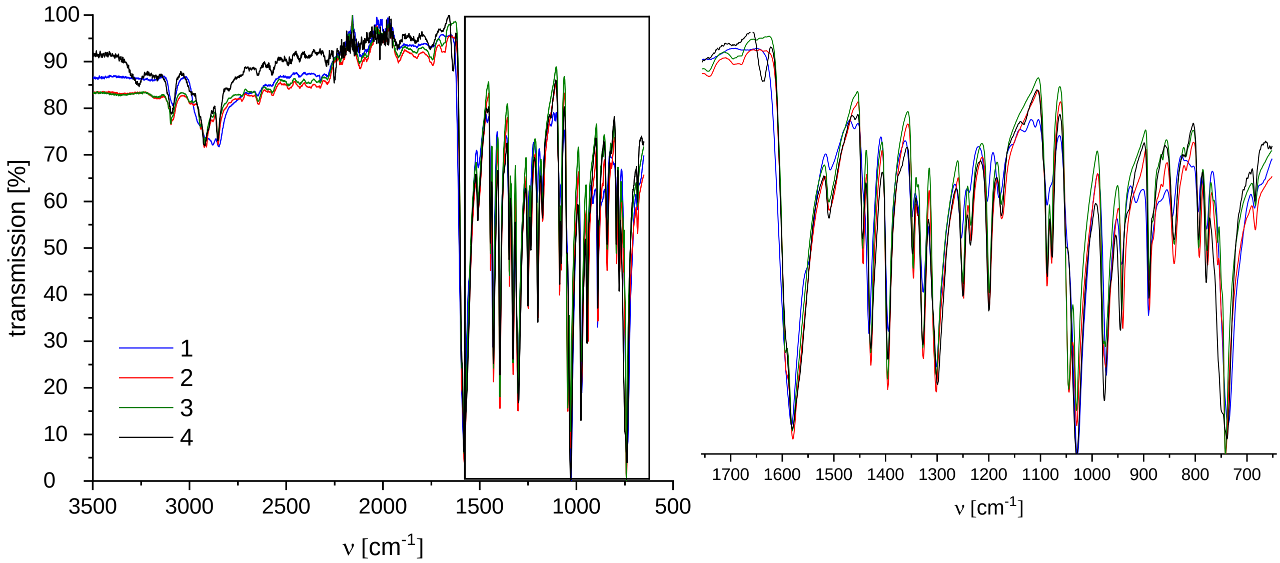

Figure 18 IR spectra of complexes **1**-**4** in full measurement range (left) and enlarged fingerprint region (right).

Table 3 Assignment of relevant IR bands of the reported complexes **1-4**.

v [cm−1]	assignment
3050-3100	$\nu_{as}/\nu_{sym\text{-}bridge}$
1580	ν_{imine}
1500	δ_{bridge} scissoring
1428	$\nu_{C\text{-}C\text{-}pyrrol}$
1369	δ_{bridge} wagging
1347	$\delta_{CH\text{-}imine}$ rocking
1240	δ_{bridge} twisting
1122	$\nu_{CH\text{-}pyrrol}$
1030	δ_{pyrrol} scissoring
810	δ_{pyrrol} wagging

6. Computational Details

All quantum chemical calculations have been performed using the TurboMole, Orca and AIMALL software packages. The technical details are given in the paper. Many online tutorials on how to actually perform QC calculations exist. One of the authors has made several such tutorials. For more information on how to perform Orca calculations and analyse the electron density, see e.g.:
https://events.prace-ri.eu/event/942/attachments/1117/2009/QC-basicTutorial.pdf
https://events.prace-ri.eu/event/942/attachments/1117/2022/CSC_2020_multiwfn.pdf

Multi-reference Calculations for Pa(IV)

When dealing with open-shell actinide systems it is important to check whether single-reference calculations like DFT are valid. In the present study exceptional QTAIM parameters were found for the Pa(IV) complex. This made it necessary to check and compare the results of the DFT calculations with NEVPT2 calculations. The NEVPT2 calculations were performed with Orca. First a DFT calculation of the Pa-pyren complex was performed with Pa(V). This removes the *f* electron and ensures, that the empty f-orbitals are the seven LUMO's. This was checked by visual inspection and with the help of the MO coefficients. The resulting wave-function was used as a starting guess for a CAS calculation with one electron in seven orbitals. The calculation was performed as a state-average over all seven dubletts. To include effects of dynamic correlation a NEVPT2 calculation was then performed and the results subjected to spin-orbit coupling. This gives a ground state that is strongly dominated by a single configuration state function. This is reassuring as it indicates that multi-reference effects will be small. However the second and third state are only 213 and 266 cm^{-1} above the ground state and will show substantial occupation at room temperature. The highest state in this calculation is 3200 cm^{-1} above the ground state and therefore not important anymore. The resulting wave-function was then used as an input for a QTAIM analysis. It should be pointed out, that this wave-function is of CAS-type only, it does not contain SO effects or dynamic correlation effects. To have a basis for comparison we also performed a Hartree-Fock calculation of the same complex and performed a QTAIM analysis. Both HF and CAS showed very similar values. This shows clearly that a single-reference treatment for the ground-state bonding properties is valid. An interesting side note is, that the QTIAM metrics for CAS and HF all show much weaker Pa-N bonding than the DFT calculation. This shows that dynamic correlation is very important for a proper description of the Pa-N bond, whereas static correlation can be neglected.

Table 4. DI and ρ values for $An–N_{pyrrolide}$ and $An–N_{imine}$ bonds in $[An(pyren)_2]$ complexes (An=Th-Pu).

	DI		ρ	
	**$An–N_{pyrrolide}$**	**$An–N_{imine}$**	**$An–N_{pyrrolide}$**	**$An–N_{imine}$**
Th	0.37629	0.32335	0.06341	0.05735
Pa	0.50758	0.43496	0.07038	0.06388
U	0.43692	0.35158	0.07093	0.05974
Np	0.43168	0.36801	0.07076	0.06279
Pu	0.4445	0.36262	0.07016	0.06110

Electron Density Difference Plots

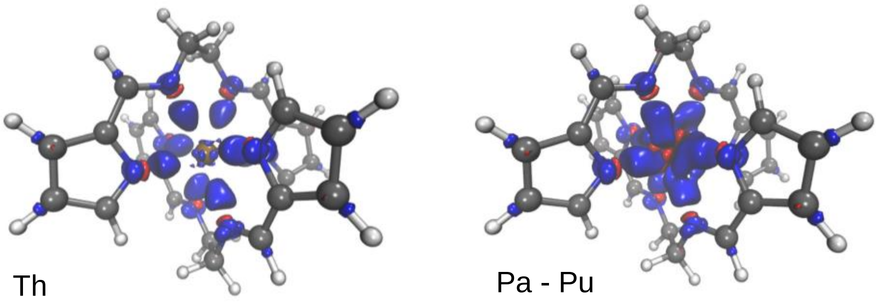

Figure 19 Electron density difference plots of the optimized [Th(pyren)$_2$] complex (left) and and [Pu(pyren)$_2$] complex (right, exemplary for An= Pa-Pu). Blue areas show increased, red areas decreased electron density.

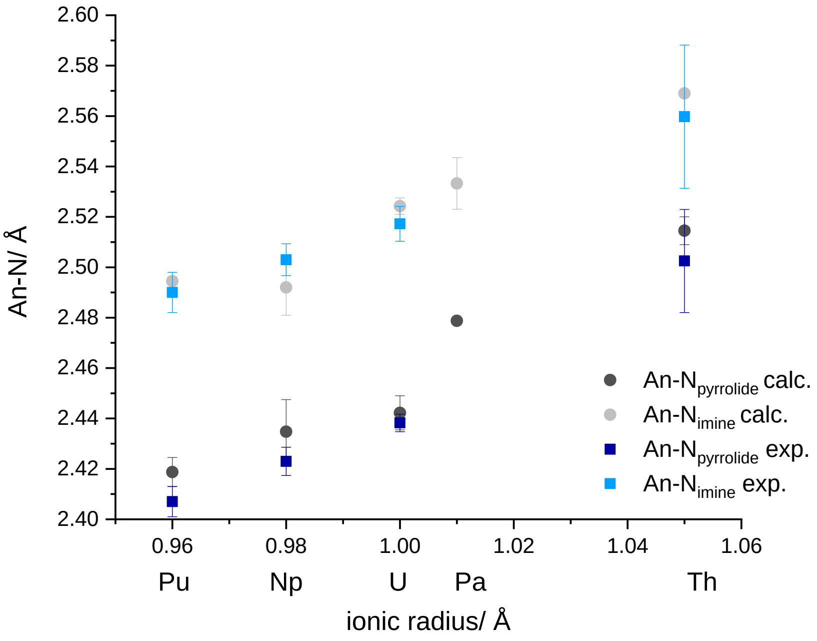

Figure 20 Plot of the experimentally determined (exp.) and calculated (calc.) An–N$_{pyrrolide}$ and An–N$_{imine}$ bond lengths against the ionic radii of the metal centre.
